# Supplementary material for: A Framework for Assessing the Impact of Outbreak Response Immunization Programs
Source: Diseases. 2024 Apr 4;12(4):73. doi: 10.3390/diseases12040073 (PMC11048879; doi:10.3390/diseases12040073)
Supplement: Supplementary file 1 [file diseases-12-00073-s001.zip › diseases-2904682-supplement.pdf]

diseases

MDPI

Supplementary Materials

Supplement A: Interview questions and results of thematic analysis

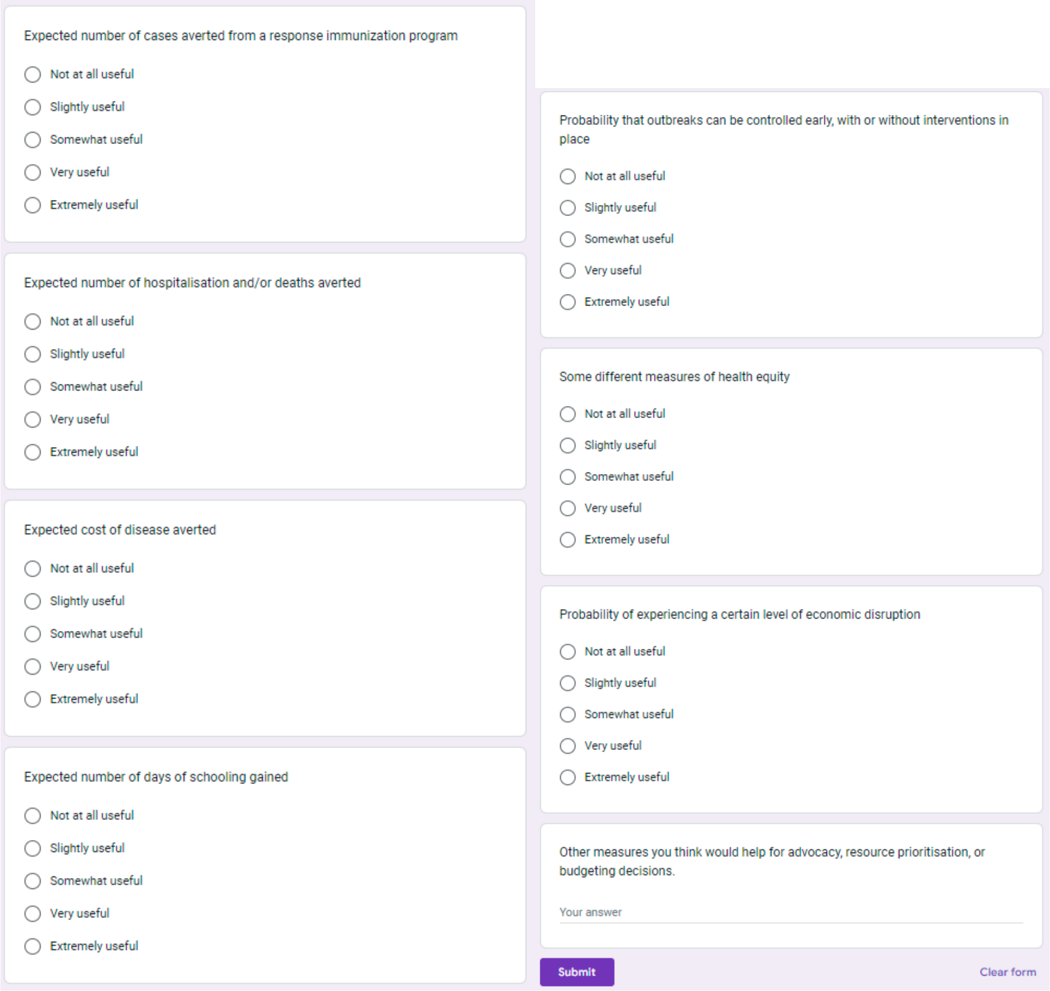
Expected number of cases averted from a response immunization program
- Not at all useful
- Slightly useful
- Somewhat useful
- Very useful
- Extremely useful

Expected number of hospitalisation and/or deaths averted
- Not at all useful
- Slightly useful
- Somewhat useful
- Very useful
- Extremely useful

Expected cost of disease averted
- Not at all useful
- Slightly useful
- Somewhat useful
- Very useful
- Extremely useful

Expected number of days of schooling gained
- Not at all useful
- Slightly useful
- Somewhat useful
- Very useful
- Extremely useful

Probability that outbreaks can be controlled early, with or without interventions in place
- Not at all useful
- Slightly useful
- Somewhat useful
- Very useful
- Extremely useful

Some different measures of health equity
- Not at all useful
- Slightly useful
- Somewhat useful
- Very useful
- Extremely useful

Probability of experiencing a certain level of economic disruption
- Not at all useful
- Slightly useful
- Somewhat useful
- Very useful
- Extremely useful

Other measures you think would help for advocacy, resource prioritisation, or budgeting decisions.

Your answer

Submit

Clear form

Figure S1. Questionnaire prompts provided to stakeholders during the interview process. Additional discussions occurred around the justification and explanation of the responders' answers during the interview, and notes were taken for thematic analysis.

Ethics: additional details

Only authors at the Burnet Institute had access to video recordings prior to filling out the data sheet. Video recordings were destroyed following completion of data sheets and notes. All data sheets and field notes will be stored for seven years according to Alfred Health guidelines, after which they will be destroyed. All electronic files were stored in a password-protected network drive at the Burnet Institute.

Methods: data extraction and thematic analysis

Ordinal response data from the interview surveys were extracted and analysed in Excel, with the frequency of the responses ("not at all useful", "slightly useful", "somewhat useful", "very useful" or "extremely useful") and the most frequent response for each output metric being recorded. Interviewees' responses to the final question were recorded and assessed as a part of the thematic analysis performed for all interviews.

Extensive notes were taken during each interview or while reviewing recordings in order to capture the details of the discussions which occurred for each survey question. These

notes were compiled and a thematic analysis was performed to identify recurring themes in the assessment of each proposed metric, how the participants' backgrounds may influence the metrics which they consider to be most useful, and to categorise any additional measures which were suggested.

Results: Interview findings on initially proposed output measures

Table A1 summarizes the strengths, weaknesses, and key ideas raised for each of the seven initially proposed output measures (which can be seen in Figure S1). In general, the most effective measures were identified as those which are easily digested, relatively consistent across settings or disease contexts, and easily framed as either a direct health or economic impact of investment in ORI.

Results: Other potential measures identified

Beyond ranking the initially proposed metrics, stakeholders were also asked to provide any other potential measures which they considered useful. Other suggested metrics to consider were disability-adjusted life years (DALYs) alongside cases and deaths, as well as some impacts that outbreaks could have on the broader healthcare system (e.g., infected health care workers, overburdened systems, disruptions to standard services). DALYs were the most frequently suggested additional measure, being suggested in three out of sixteen interviews, with most other measures only being suggested once.

Results: thematic analysis

Cases and deaths averted were typically seen as the most useful, as they were easy to interpret and consistent across settings, but it was noted that cases averted may have less meaning for pathogens with lower severity. Hospitalizations averted was almost always noted as a separate category, due to the dependency on case definitions and health system capacity.

Many stakeholders found economic outcomes favourable, particularly those working with Ministries of Finance, company Board Members or others who are required to consider financial outcomes to support their decisions. However, challenges were noted when using economic outcomes to measure potential impact, including variations in costs, health system functions and service availability across settings, and difficulties in measurement due to limited cost or economic data relative to epidemiological data.

Stakeholders were most divided on outcomes related to timely cessations of outbreaks. For some stakeholders this or related measures could be used to quantify the impact of investment towards strategic targets or major performance measures of organizations, however for others the measures were seen as too complex to communicate in high-level meetings with time-constraints.

Secondary health impacts were also considered important, such as increased poverty, delayed health outcomes or health seeking behaviours, or worsened mental health as a result of large, prolonged outbreaks, but it was acknowledged that these factors can be difficult to measure and may also be inconsistent across settings and pathogens.

Multiple stakeholders mentioned the potential impacts of outbreaks on migration and cross-border travel, with multiple rationales. It was noted that outbreaks can have impacts on migration and economic consequences for labor migrants or those who need to travel for work. More broadly, outbreaks could significantly impact the economy of a country if international travel warnings were issued.

Table S1. Strengths, weaknesses, and key themes for the seven initially proposed output measures extracted from interviews with stakeholders.

Measure	Strengths	Weaknesses	Summary
Cases averted	Common and familiar measure<br>Recognizable and expected	Potentially less convincing for less severe diseases as many infections may be mild and considered less important<br>Difficult to compare across diseases	Useful for almost all stakeholders and an expected measure as reduced transmission is a primary effect of vaccines. However, it is contextual to the severity of the disease and the aims of the strategy being applied (i.e., elimination vs suppression).
Hospitalisations and deaths averted	Deaths averted is always expected<br>Hospitalisations are useful for estimating health system utilisation and costing	Require clear and accurate case attribution<br>Hospital data not always readily available	Considered very or extremely useful by almost every interviewee. Deaths were considered the more useful of the two measures, and strong feedback that both measures speak to different things.
Expected cost of disease averted	Donors interested in investment and ROI<br>Economic impacts can be a significant motivation	Less useful for comparing across settings as health system costs differ so much<br>Highly dependent on data availability and quality<br>Costs may be low where services are minimal<br>Cost impacts can be seen as small by governments when considered on a country scale	Received mixed responses. It was often considered very or extremely useful, however it was also seen as highly context dependent, less tangible, or requiring more specific definition.
Probability of economic disruption	Classically used in finance depts and helps development stakeholders make the case internally<br>For COVID this was a	Could be difficult to quantify<br>Difficulty in constructing consistent and useable measure across countries	Typically considered very useful as it would be well understood by stakeholders, however there were noted concerns about methods and the level of data availability.

	primary driver for decision makers<br>May apply more universally than health system costs averted		
Days of schooling gained	Measure of indirect costs and impacts beyond health	Only considering a very specific subset of population	Not considered particularly useful by most responders, and many VPDs impact younger children or whole of population. Suggestions that broader measure of working days lost/absenteeism would be more valuable.
Probability of endingoutbreak early	Core indicator for Gavi (e.g., timeliness of suppression/elimination) and other global targets<br>Plays to people's fears; powerful communication tool	May be difficult to communicate to some stakeholders (e.g., board members)<br>Potentially only useful in conjunction with something else e.g., 'saving XX lives'	Received mixed responses. Where it was clearly understood most responders considered it to be very useful, however some interviewees considered it to be confusing or not clear enough to be useful. A key point was raised was that this formed a potential link to strategic targets.
Measures of health equity	Important message in Gavi advocacy<br>Important factor to consider in public health and helps galvanise support from stakeholders who hold this as a key topic	Dependent on the specific measures of equity used<br>May divert attention away from other outcomes that are the primary aim of an ORI program	Typically, both important and useful, particularly from Gavi's perspective as they can be directly aligned with specific goals and targets. The most common suggestion was zero dose children. Consideration of regional equity may be required.

Supplement B: Description of data search strategies

Eligibility criteria for sources were:

- *Population*: Low-or-middle-income countries experiencing an outbreak of a vaccine preventable disease
- *Intervention*: Any or none
- *Control*: Controlled or not controlled
- *Outcome*: Any outcome that could potentially be used as an input measure for an infectious disease model
- *Study design*: Observational / field studies

The search was performed in three parts, the first being for epidemiological measures reported during outbreaks of VPDs, the second for measures of health system impacts and costs incurred during outbreaks, and the third being for ORI program usage data. The searched VPDs focus primarily on diseases with frequent outbreaks (such as measles and Ebola), diseases for which Gavi stockpiles vaccines (Ebola, yellow fever, meningitis, and cholera), and 'other' VPDs (i.e., mumps, pertussis, polio, hepatitis A, diphtheria). The first (epidemiological) search was conducted for all the above diseases as it was expected to have the most available data. The subsequent searches focused only on measles, Ebola, yellow fever, meningitis and cholera, based on the relative lack of discovered epidemiological data for the 'other VPDs'.

All sets of searches were conducted in English using *Google* and *Google Scholar*, with the search for epidemiological data consisting of all combinations of the terms from Table S2, in the format "[*Vaccine-preventable infection outbreak term*] [*Outbreak term*] [*Format term*] [*Region term*]". For example, "Measles outbreak report Africa" or "Ebola cluster brief South America" were among the phrases searched. Similarly, the search for health system impact and cost data was conducted using all combinations of the terms from Table S3: "[*Vaccine-preventable infection outbreak term*] [*Outbreak term*] [*Health system impact & cost term*] [*Region term*]". Finally, the third search for measures of ORI program usage and costing was performed for all combinations of the terms from Table S4: "[Vaccine-preventable infection outbreak term] [*Outbreak term*] [*Program usage and costs term*] [*Format term*] [*Region term*]". For Ebola the region term was limited to only "Africa" when searches were performed due to the lack of outbreaks in other regions, and similarly the search scope for yellow fever was reduced to "Africa" and "South America" in all searches.

Returned results were examined, and the source recorded if it included relevant data for an outbreak of a VPD in an LMIC setting. Where searches returned relevant data for more than one outbreak - either within a single report or brief, or as a collection of sources - all relevant sources were recorded. Where a search returned a source that was directly relevant to the terms used it was considered 'primary'. For example, if a search for "Measles outbreak report Africa" returned a site which compiled multiple outbreak reports in African nations then any reports of measles outbreaks would be considered primary sources, but any listed reports of outbreaks of other diseases (i.e., not directly matched to the search terms used) would be considered 'secondary' sources.

There was a notable difference in the types of results returned when comparing the different parts of the search. The search for epidemiological data typically returned government reports and other grey literature, as expected, but the searches for health system impact and cost measures, and ORI program data returned primarily academic articles.

Table S2. Set of terms used for data availability search of vaccine preventable disease outbreak reporting of epidemiological measures.

Vaccine preventable infection outbreak	Outbreak	Format	Region
Measles	Outbreak	Report	Africa
Ebola	Cluster	Brief	Asia
Yellow fever			South-East Asia
Meningitis			South America
Cholera			Pacific
Mumps			*Country specific
Pertussis			

Polio			
Hepatitis A (HAV)			
Diphtheria			
Disease			

Table S3. Set of terms used for data availability search of vaccine preventable disease outbreak reporting of health system impact and cost measures

Vaccine preventable infection outbreak	Outbreak	Health system impact & cost	Region
Measles	Outbreak	Health service delivery	Africa
Ebola	Cluster	Essential medicines	Asia
Yellow fever		Economic impact	South-East Asia
Meningitis		Cost	South America
Cholera		Vaccine cost	Pacific
			*Country specific

Table S4. Set of terms used for data availability search of vaccine preventable disease outbreak reporting of ORI program usage and costs

Vaccine preventable infection outbreak	Outbreak	Program usage and costs	Format	Region
Measles	Outbreak	Immunization response	Report	Africa
Ebola	Cluster	Vaccine response	Brief	Asia
Yellow Fever				South-East Asia
Meningitis				South America
Cholera				Pacific
				*Country specific

Additional country-specific searches

During all searches for data measures, the key identified sources primarily represented outbreaks in African nations, and relatively fewer results were found for LMICs in other regions. In order to find additional data for other regions, or verify that additional sources were not available, a variation of the search strategy was performed. The search was expanded to include the combinations of the non-region terms and the top 10 most populated LMICs from both Asia and the Pacific, and South America.

Analysis of extracted results

The frequencies with which each recorded variable was captured for measles, Ebola, yellow fever, meningitis, cholera and 'other VPDs' were tabulated. Results have been analysed separately for epidemiological data, economic/cost and health system-related data, and ORI program usage data. The broader search for 'other VPDs' was performed only for the epidemiological data; it was performed for additional context around data availability, but the primary focus of the searches were for measles, Ebola, yellow fever, meningitis, and cholera.

Supplement C: Reported measure frequencies from each data search

Review findings: epidemiological data

Cumulative cases (probable and confirmed), cumulative deaths, and case fatality rate (CFR) were the most reported metrics for Ebola, reported in at least seven of the ten sources found (Figure S2).

For measles (Figure S3), the most reported measures were again cumulative cases and cumulative deaths, occurring in at least 25 out of 31 sources, cumulative cases per district being reported in 16 sources, and both cumulative probable cases and cumulative cases by age group being reported in 15.

Eight sources were found for yellow fever (Figure S4), and in at least five of them the cumulative cases (probable and confirmed) were reported, therefore being the most common measures. For cholera, there were six sources found over the search (Figure S5), and in all six the cumulative deaths and cumulative cases were reported. Only three data sources were found for meningitis, and therefore the results have not been plotted, but cumulative confirmed cases and case fatality rate were reported in all three sources.

For the other reported VPDs (excluding measles, Ebola, yellow fever, meningitis, and cholera), cumulative cases, cumulative deaths, and case fatality rate were again the most frequently reported data, appearing in at least six out of 13 sources found (Figure S6).

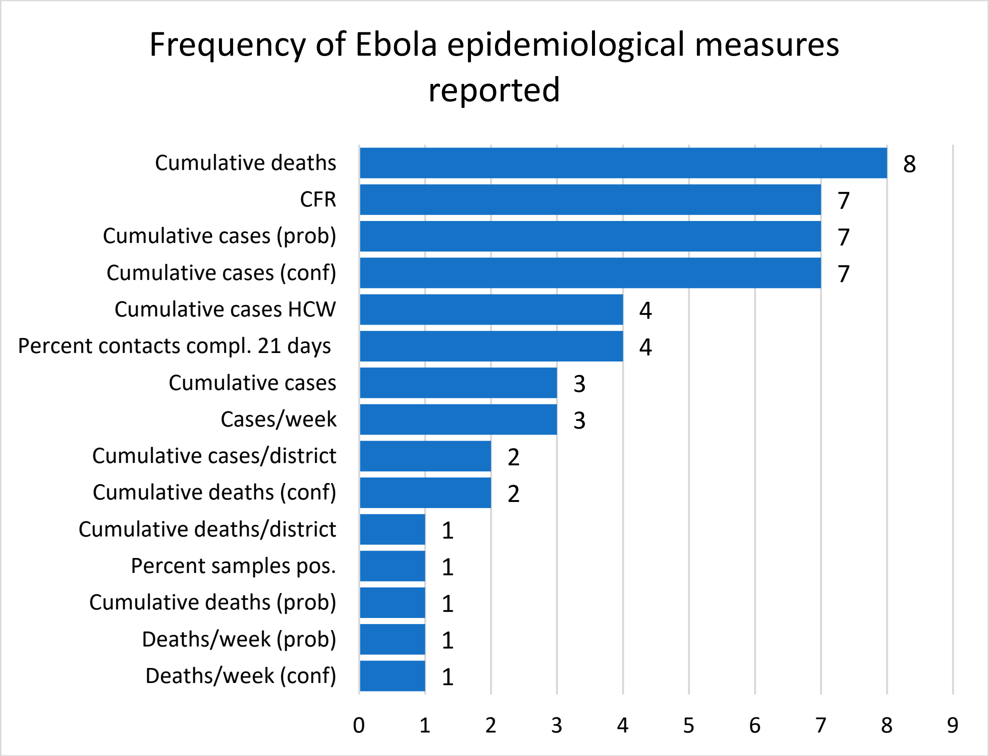

Figure S2. Frequency of reported metrics for outbreaks of Ebola

*Prob, conf, compl., and pos. are abbreviations for probable, confirmed, completed, and positive.

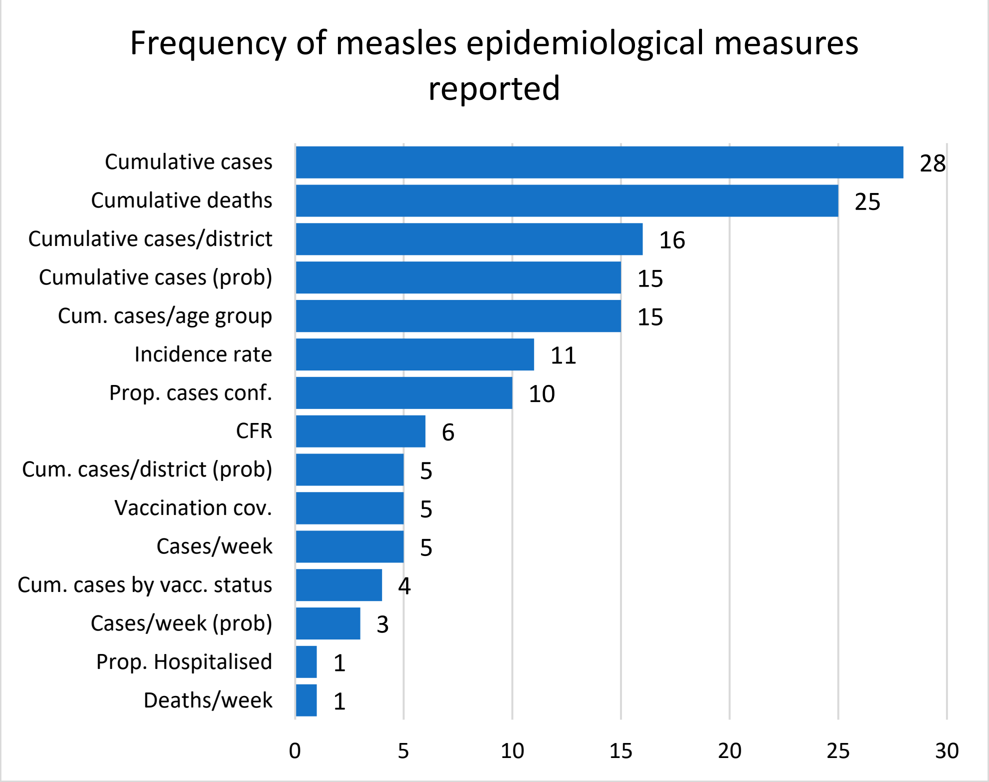

Figure S3. Frequency of reported epidemiological metrics for outbreaks of measles *Cum., prop., cov., and vacc. are abbreviations for cumulative, proportion, coverage, and vaccinated.

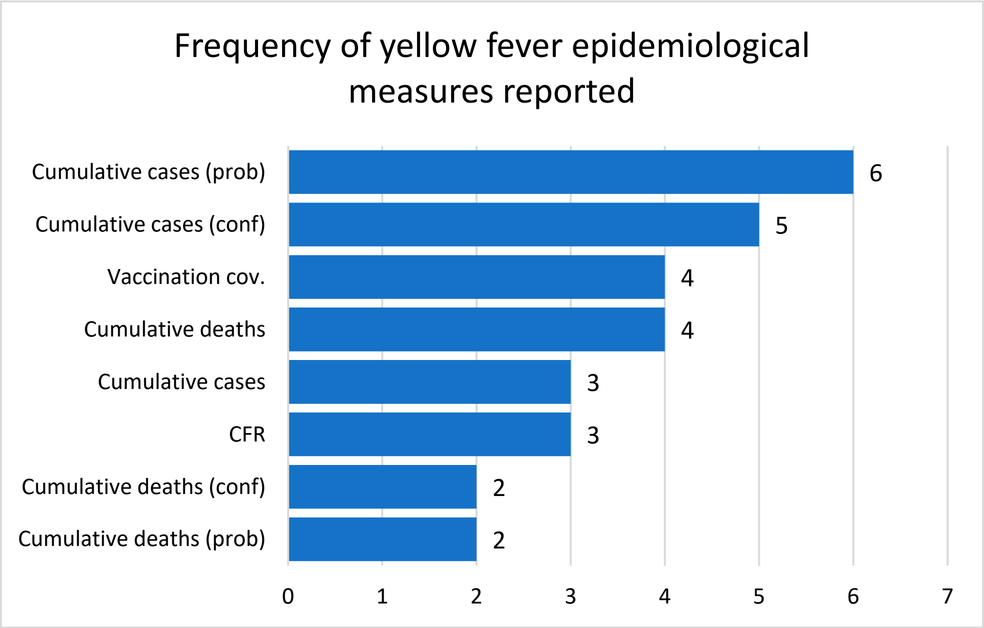

Figure S4. Frequency of reported epidemiological metrics for outbreaks of yellow fever

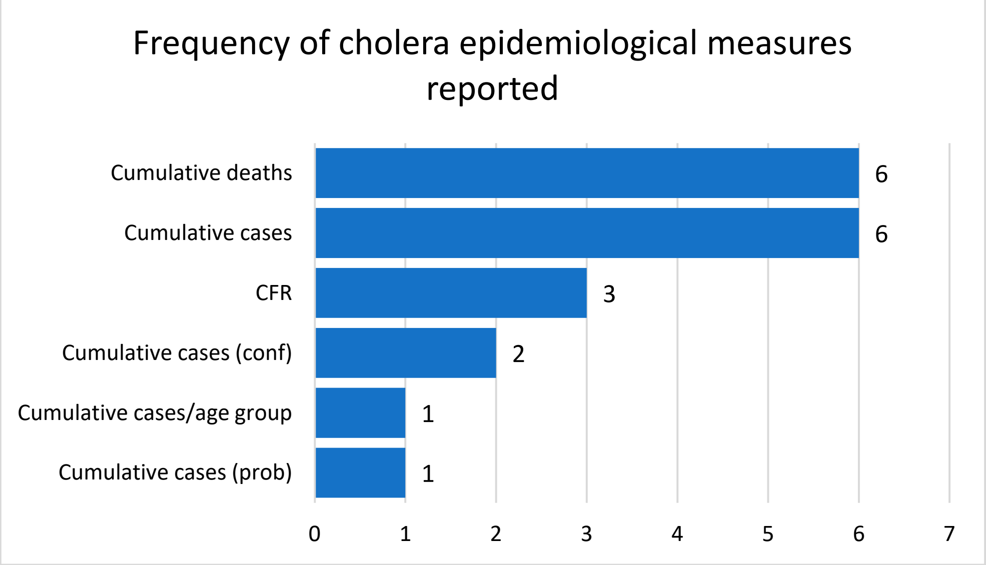

Figure S5. Frequency of reported epidemiological metrics for outbreaks of cholera

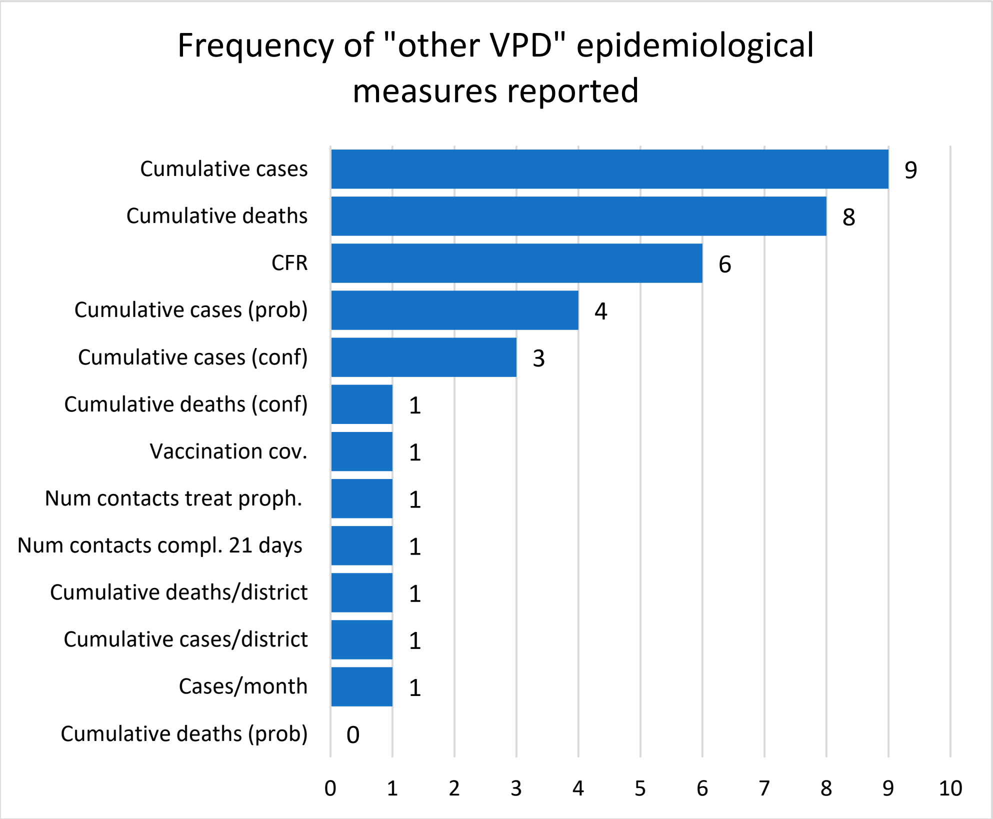

Figure S6. Frequency of reported epidemiological metrics for outbreaks of "other VPDs" (excluding measles, Ebola, yellow fever, meningitis, and cholera)

*Num and proph. are abbreviations for number and prophylaxis.

Review findings: economic/cost and health system-related data

The most frequently reported measure for health system and economic impacts due to outbreaks of Ebola was the change in number of visits to health care facilities and hospitals during an active epidemic period, found in 9 out of 21 articles (Figure S7). Most sources found were for relatively narrow analyses, typically considering impacts on pregnant women, people living with HIV, or people infected with malaria. As such, there were a

broad range of measures found, but most were only reported in a single article. There were also only a limited number of sources reporting on the economic impacts of Ebola outbreaks, and where reported the impacts were very broad scale estimates of impact on the country's overall GDP or GDP growth. The search conducted for measles returned six relevant data sources, all of which were focused on economic impacts, with the most frequently reported measures being the direct and indirect costs associated with the outbreak (Figure S8). Here direct costs refer to costs directly associated with the health system impact of the outbreak, e.g., expenditure on disease management, healthcare workers (HCW) salaries, and hospital bed usage, whereas indirect costs refer to broader economic impacts from the outbreak, outside of the health care system, such as overall GDP or GDP growth.

The findings of the search for yellow fever, meningitis, and cholera are similar to the findings of the search performed for measles. The most frequently reported metric for meningitis was again direct health system costs, reported in nine out of 11 sources, with indirect costs being the next most reported, in five sources (Figure S9). For cholera, vaccine costs were reported most frequently in 12 out of 16 sources, and both direct and indirect costs were reported in 10 (Figure S10). No sources were found for yellow fever in this search.

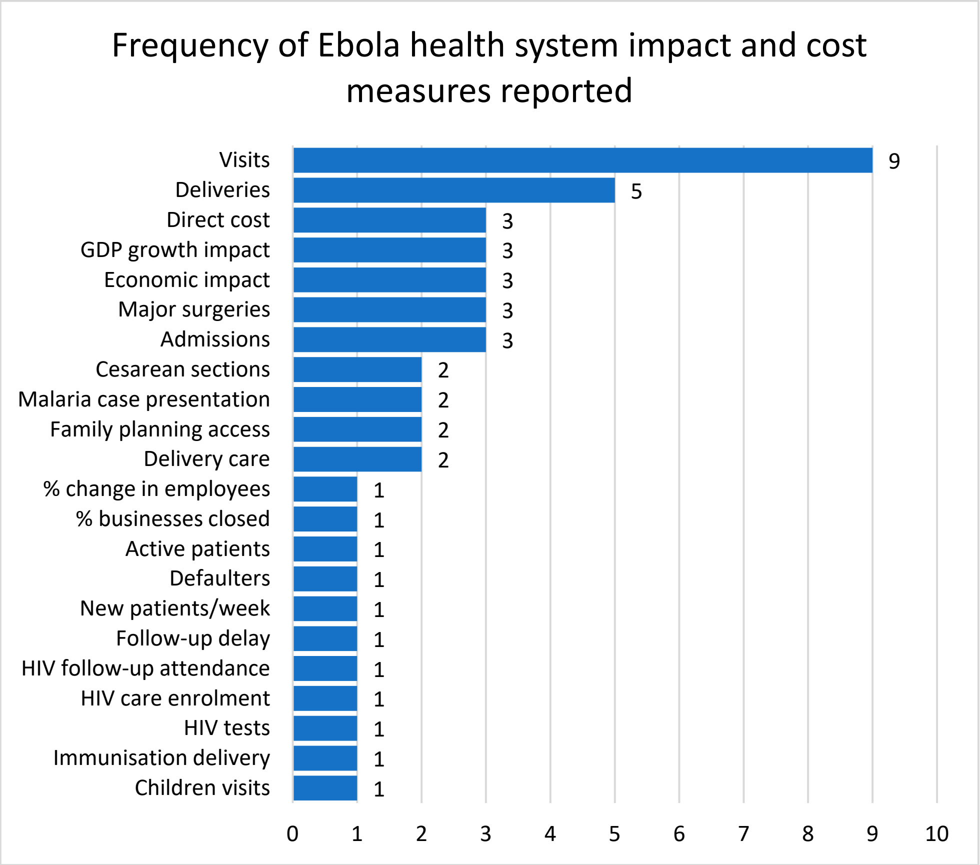

Figure S7. Frequency of reported metrics for health system and economic impacts due to Ebola

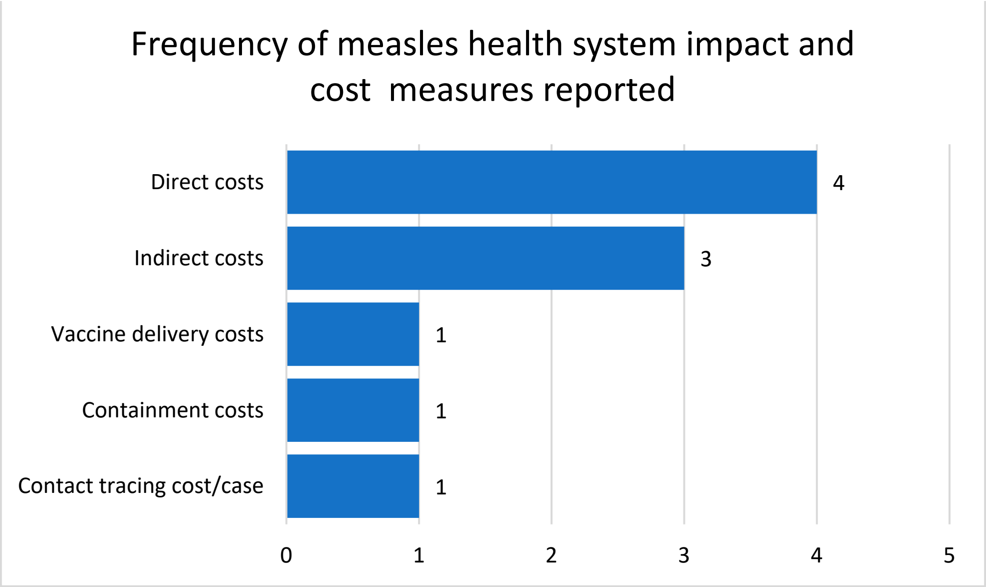

Figure S8. Frequency of reported metrics for health system and economic impacts due to measles

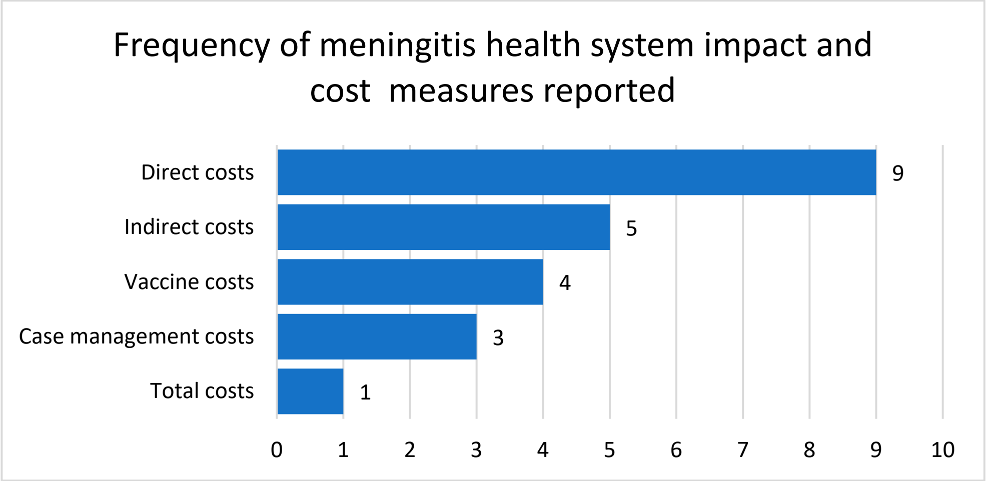

Figure S9. Frequency of reported metrics for health system and economic impacts due to meningitis

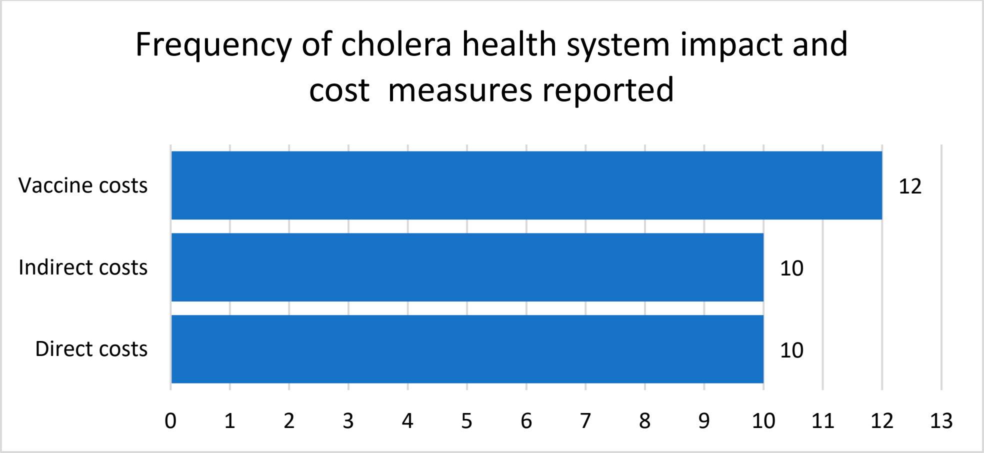

Figure S10. Frequency of reported metrics for health system and economic impacts due to cholera

Review findings: ORI program usage data

There was little reported data found around the implementation of ORI programs for outbreaks of Ebola, with only five sources found. This is likely due to the relatively recent development of the Ebola vaccine, with the first trials being conducted in 2014-2016 during outbreaks in Western Africa. The total number of people vaccinated was reported in all five sources, and three of the five sources also reported total HCWs vaccinated as a part of the response and the number of known contacts of cases which were vaccinated as a part of ring-vaccination strategy (Figure S11).

Response program data for measles outbreaks appears to be more frequently reported, with the search returning 14 sources, however the scope of the metrics reported is quite shallow. In 12 out of the 14 data sources the total number of individuals vaccinated as a part of the outbreak response was the only metric reported. Measures such as the wastage or the time taken to begin a response were only found once (Figure S12).

The search for cholera returned eight sources (Figure S13), with the total number of people vaccinated by the response again being the most frequently reported data, appearing in four of the sources. The second most common measure, appearing in three sources, was the time taken to begin a vaccine response. The search returned five results for yellow fever; however, they all only reported the total number of people vaccinated and no other measures (figure not shown). In contrast, only two sources were found for meningitis which reported four metrics, of which only the time taken to begin a response appeared in both sources.

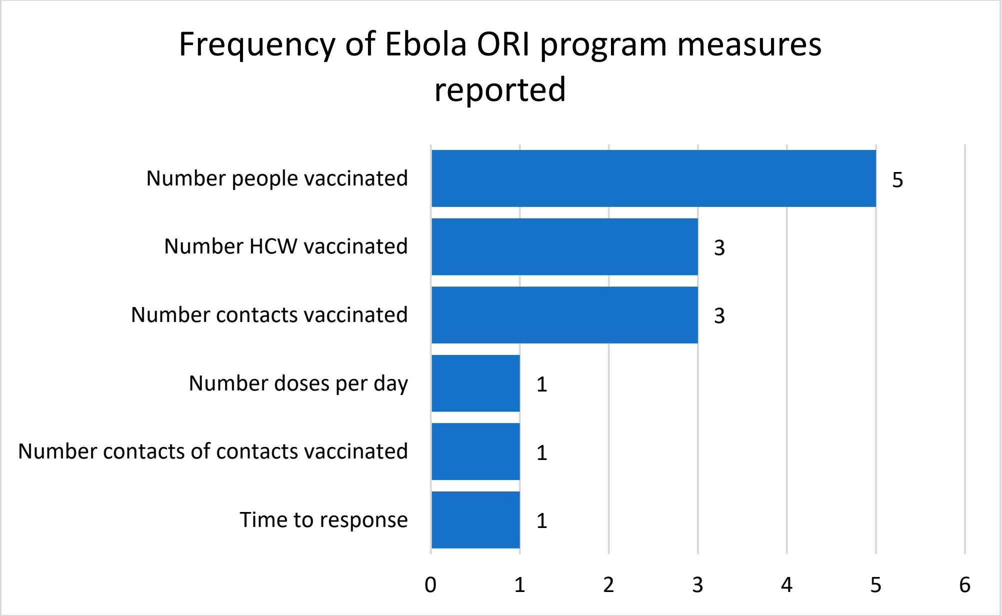

Figure S11. Frequency of reported metrics for ORI program usage data due to Ebola

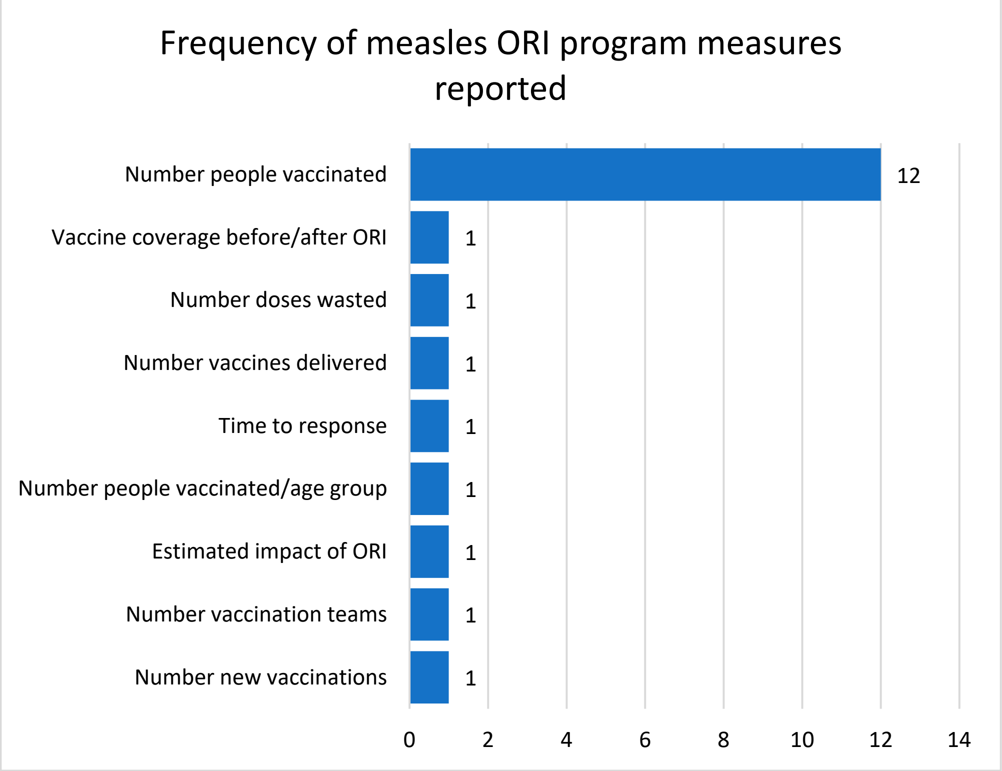

Figure S12. Frequency of reported metrics for ORI program usage data due to measles

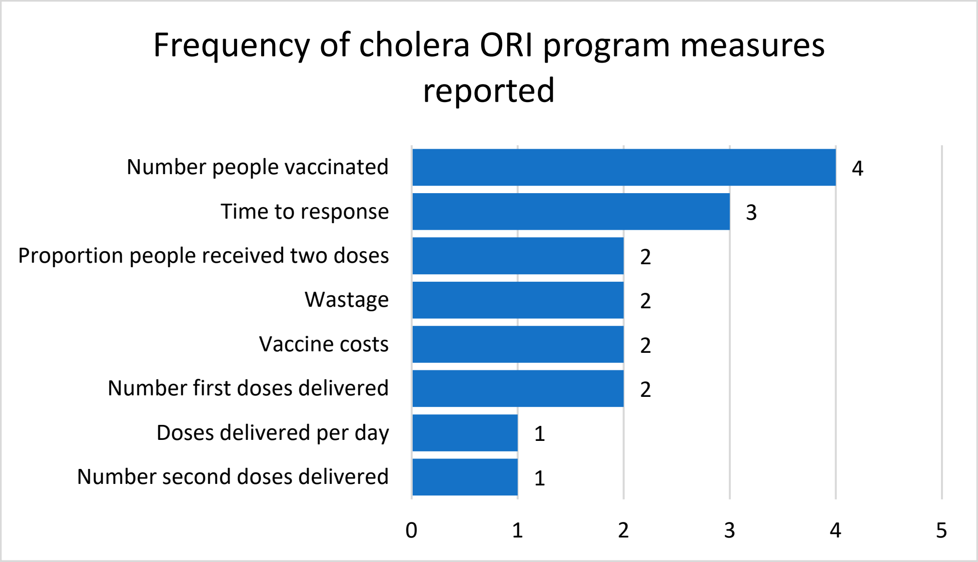

Figure S13. Frequency of reported metrics for ORI program usage data due to cholera

The full list of sources retrieved during this search is detailed in the 'Review sources' spreadsheet, aggregated for the epidemiological measures, health system impacts and costs incurred during outbreaks, and ORI program usage measures.

Supplement D: Further description of scenario categories

Baseline scenario

To evaluate the impact of past investments, the baseline scenario used would typically be a modelled simulation of an existing outbreak, fit to the empirical data. Depending on the pathogen and setting, in this baseline scenario an outbreak response may or may not have

been implemented, other interventions (e.g., test, trace and isolate or stay-at-home orders) may or may not have been in place, and the outbreak may or may not have been successfully ended.

When considering the value of future or additional investments, it may be appropriate to use a baseline scenario that assumes a continued status-quo with an existing ORI, which can be compared to additional investments in some domains, or to use a scenario with no ORI program in place if one does not already exist in the setting.

Scenarios with improved scale of response

Investments in healthcare worker readiness, healthcare system capacity, cold-chain logistics, reducing wastage, and vaccine stockpile size can be considered to increase the proportion of the population who can be reached as part of an ORI program during an outbreak. The connection between amount of investment and the improvement in program reach will depend on pathogen and setting and will need to be estimated independently for any ORI investment case. The relationship between investment and improved coverage will be a model input assumption, estimated from data on past ORI spending and responses.

The modelled scenarios would involve varying the proportion of the population who are immunized as part of the ORI program during an outbreak, assuming a fixed rate of delivery, and comparing outcomes. For example, a set of scenarios could consider investing such that 20%, 40%, 60%, or 80% of an affected population can be reached during an outbreak. The results could be compared to a counterfactual of no ORI program, or against each other to estimate the incremental impacts of additional investment in program scale.

A related type of scenario would involve modelling variations in the initial vaccine coverage before an outbreak. For some settings and pathogens, it is possible that sufficient initial vaccine coverage could enable an ORI program to easily achieve herd immunity with little additional coverage during an outbreak. Using modelling to determine how sensitive an ORI program is to the initial vaccine coverage could inform investment priorities.

Scenario with improved speed of response

Investments in healthcare worker training and readiness, cold-chain logistics, testing and surveillance, and vaccine stockpile logistics can be considered to increase the speed at which an ORI program is initiated or delivers vaccinations during an outbreak. The connection between amount of investment and the improvement in vaccination commencement and speed will depend heavily on pathogen and setting, and will need to be estimated independently for any ORI investment case.

The modelled scenarios would involve varying the time taken to begin an ORI response or the rate at which vaccines are delivered to the population as part of the response, assuming a fixed achievable coverage, and comparing outcomes. For example, a set of scenarios could be designed in which the average time taken to detect a case and begin the immunization response is varied by one-week intervals, from two months to one week. The outcomes of each scenario could be compared to estimate the incremental impacts of additional investment in early detection mechanisms.

Alternatively, a set of scenarios could be modelled in which the number of vaccines delivered by the program each day is varied (up to some threshold capacity for population coverage). The results of each scenario could be used to estimate the incremental impacts of investment in improved program delivery speed.

Scenarios with improved population prioritization
Investments in outreach, education, access to vulnerable populations, healthcare worker training, and contact tracing capacity and logistics can be considered to increase the capacity to prioritize vulnerable populations or target contacts of known cases during an outbreak. The connection between amount of investment and the improvement in prioritization/targeting will depend heavily on pathogen and setting, and will need to be estimated independently for any ORI investment case.

These types of scenarios would be quite setting and pathogen dependent, but could provide insight into useful response strategies in certain contexts. For example, comparing an Ebola Virus Disease outbreak scenario with vaccines targeted at contacts of known cases against a scenario with random vaccine delivery could provide insight into how impactful investment in contact tracing as part of an ORI program may be.

Scenarios that compare prioritized vaccine delivery to vulnerable populations (such as young children, older people, or migrants) against a baseline scenario with random delivery could be used to estimate the impact of investing in program outreach, access, or education to relevant vulnerable communities.

Scenarios with combined or time-varying parameters
The specific quantitative relationship between investment and parameter variations must be estimated for each context. Each parameterization defines a different scenario, which can be modelled to produce a standard set of outputs that can be compared against each other. However, this parameterization does not need to be static, and depending on the aim of the evaluation analysis, scenario types could be combined or parameters may be set to vary with time. For example, general investment in planning may improve time taken to begin a response AND coverage AND response speed. It may also be useful to estimate the tradeoffs in prioritizing speed over coverage, or to model a scenario in which there is an initially rapid and targeted delivery of vaccines which then shifts to a slower and broader response over time. This flexibility will allow for the framework to evaluate more complex questions and provide more detailed information to stakeholders.

Supplement E: Framework phase breakdown and example decision points

Problem framing and data sourcing

<u>Choose disease and setting context</u>

- What is the disease(s) of interest;

- What is the geographical scope and time frame to be considered (e.g., district/province/country/region; single outbreak versus multiple outbreaks; start/end date of analysis);

- Which elements of the ORI are within the scope of analysis (e.g., within-country activities only; for integrated service delivery only some components).

<u>Identify key stakeholders and objectives</u>

- Who is the analysis for, and who are other key stakeholders (e.g., implementation partners, government, donors);

- What will the results be used for once available (e.g., understand which ORI components are the most impactful; influence public health policy; inform resource allocation decisions);

- Whose perspective should the analysis take to best meet stakeholder needs;
- Will the analysis consider retrospective or future investment outcomes (e.g., aiming to quantify the benefits of past investment on specific events that occurred; or to make a case for maintaining or expanding investments in the future; or to estimate the benefits of implementing an ORI program in a new setting).

Decide scenarios to model

- What data is available to inform relationships between investment and ORI program operations (e.g., investment mapped to coverage, speed, targeting);
- Based on data availability, which model parameters can be used to define scenario(s);
- From these parameters, which scenario(s) could be created to best align with stakeholder needs and objectives;
- Which parameters will be changed and by how much, for each proposed scenario;
- What expert advice could help inform relevant scenarios.

Select required outputs

- What types of outputs are feasible with the level of data availability in the setting;
- What types of outputs (e.g., health, economic, risk and disruption) will be most useful for stakeholders to meet their objectives.

Model choice

Determine minimum model requirements

- For the chosen scenarios, outputs and expected level of data availability, are there existing fit-for-purpose models;
- What type of model is most suitable (e.g., statistical vs mathematical; compartmental vs agent-based; deterministic vs stochastic);
- What minimum stratification does the model require (e.g., age-groups, at-risk populations);
- How should transmission modes be captured (e.g., appropriate approximations for person-to-person, environmental, vector-borne transmission);
- What minimum level of detail is required in the program implementation;
- Will additional programs be required in the model (e.g., existing vaccination programs);
- How will model complexity influence results interpretation and communication;
- Will there be an economic component to the model.

Parameterise model inputs from available data

- Is there sufficient data available to parametrize the epidemiological/demographic/programmatic parameters of the model;
- Are the data of sufficiently high quality that their use in the model is justified;
- Can the model be simplified or expanded so that it aligns with the data;
- If issues in parameterization arise, can they be resolved by reconsidering the scope of the desired scenarios or outputs;
- What other assumptions are required within the model, and how are they informed.

Model implementation

Calibrate model

- Which parameters are constrained by data, and which are determined through calibration;
- Does the model reproduce the observed data (such as daily or cumulative cases and deaths);
- What parameters are the most/least sensitive in the model;
- How robust is the model calibration (e.g., can the same model fit be achieved by other parameter combinations);
- Are the fitted parameter values within plausible ranges.

Model scenarios

- Using the calibrated models as the baseline scenario, what happens when the model is run with different parameter combinations for the pre-defined scenarios.

Interpretation and communication

Analyse results

- Are differences in scenarios as expected/sensible, and if not are they explicable;
- How do the results align with previous studies;
- What summary data and/or plots will best represent the outputs;
- Are additional analysis steps required (e.g., transformation of health measures to DALYs; combining economic inputs/outputs);
- Is it more effective to present results in different ways (e.g., consider the relative or absolute differences between scenarios).

Interpret and communicate results

- What are the key messages and how can the results be explained simply;
- What data, parameters or model features are driving the results;

- What is the uncertainty in results;
- How sensitive are the results to any model assumptions or data inputs;
- Do results support or present a contrary view to stakeholder expectations;
- What are the implications of the results, and how generalizable are they;
- How can the results be linked back to the initial objectives and stakeholder needs.

Supplement F: Detailed 2014-15 Madang Province, Papua New Guinea measles analysis

Measles outbreak in Madang Province, Papua New Guinea
This section details the analysis performed for the measles outbreak in Madang Province, Papua New Guinea (PNG). The objectives were to estimate the return-on-investment of the ORI program, in a way that is useful for government and international stakeholders to support continued financing of these programs in PNG.

Background
In June 2014 a measles outbreak was declared in PNG and remained active until March 2015. The outbreak caused a total of 11,097 reported infections, 5,073 of which occurred in Madang Province, which will be the focus of this study. Madang Province also recorded 30 deaths among the cases, and in the years preceding the outbreak had a measles vaccination coverage of 38%, which was significantly below the national average of 65%[34,39]. During the outbreak there were a reported 160,460 vaccine doses issued, but only 71,474 doses administered, with a wastage rate of 55%[34].

The objectives of this analysis were to estimate the return-on-investment of the ORI program, in a way that is useful for government and international stakeholders to support continued financing of these programs in PNG. The analysis also considered the impacts of targeted improvements to key aspects of the response, to identify the highest priority elements of the response (e.g., speed versus coverage) and where the greatest gains could be in advance of or during a future measles outbreak.

Aims
<u>Aim 1</u>: estimate the cumulative infections, deaths, disability adjusted life years (DALYs) averted by the ORI program.

<u>Aim 2</u>: estimate the economic costs averted by ORI (health system costs of cases and economic burden of deaths and morbidities).

<u>Aim 3</u>: identify elements of the response (speed of rollout, coverage, time to commencement, baseline vaccine coverage) that have the greatest influence on ORI program impact.

Methods

<u>Data</u>
Data availability for this setting is limited. The only publicly available indicators identified were the start and end dates of the immunization response, the baseline vaccine coverage, the total number of cases, deaths, and vaccine doses delivered over the outbreak period, and an estimate of vaccine wastage during the response[34].

Model

Due to the scarcity of data for this outbreak, a simple model was required. A stochastic susceptible-exposed-infected-recovered (SEIR) compartmental model with vaccination, built in the Atomica framework[40], was used (represented schematically in Figure S14). People begin in the susceptible or vaccinated compartment, based on initial vaccine coverage in Madang Province. Both susceptible and vaccinated people can become infected at a rate that is proportional to dynamic prevalence, however vaccinated people have reduced risk due to vaccine protection. Following infection, people have an incubation period before becoming infectious, and after an average duration of infection can either die or recover.

The model's stochastic implementation meant that each time step (representing a day), integer numbers of people were moved between compartments, sampled from binomial distributions with the corresponding rate parameters (see Table S5).

The duration of vaccine immunity was set to well beyond the scope of the model period as it is assumed that no waning of immunity effects would be relevant over the outbreak period. Table S5 details the parameters used within the model.

As there was insufficient data to inform age or risk-based transmission, the model consisted of a single population representing Madang Province, which was assumed to be composed entirely of either susceptible or vaccinated people (with 38% coverage) before the outbreak. The outbreak response began in June 2014, but it is unclear when the initial cases entered the community. As measles is endemic in many low-and-middle-income-countries (LMICs) it is expected that there may have been some transmission ongoing for a significant time before the response began, with the response being triggered by a significant growth in burden. In order to capture a period of growth before the vaccine response is known to have started the model was initialised in April 2014 with a background prevalence of approximately 200 cases.

Table S5. Model parameter values and sources.

Parameter	Value	Source
Average duration of exposed period	8 days	Centers for Disease Control and Prevention[41]
Average duration of infection	11 days	Centers for Disease Control and Prevention[41]
Vaccine protection against infection	96·7%	Pillsbury and Quinn[42]
Madang Province population size	493,904	National Statistical Office of Papua New Guinea[43]
Madang Province underlying vaccine coverage	38%	Kamac et al[34].
Number of doses delivered during response	71,474	Kamac et al[34].
Cost of response	US$454,292*	Kamac et al[34].
Disability weight for measles infection	0·152	Estimate from Stein et al[44].
Average life expectancy in PNG	64·5 years	The World Bank[45]

Average age of death from measles	4 years	Assumption, as most measles deaths are in children under 5[46].
Proportion of measles cases requiring hospitalisation	25%	Assumption, as 1 in 4 measles case are estimated to require hospitalisation[47].
Duration of hospital stay	5 days	Chovatiya and Silverberg[47]
Statistical value of a life year	US$4,821·43*	Calculated as 1·75 times the Gross Domestic Product (GDP) per capita for PNG, based on Stenberg et al[48].

*Inflated to 2022 US$. Source does not contain costing breakdown.

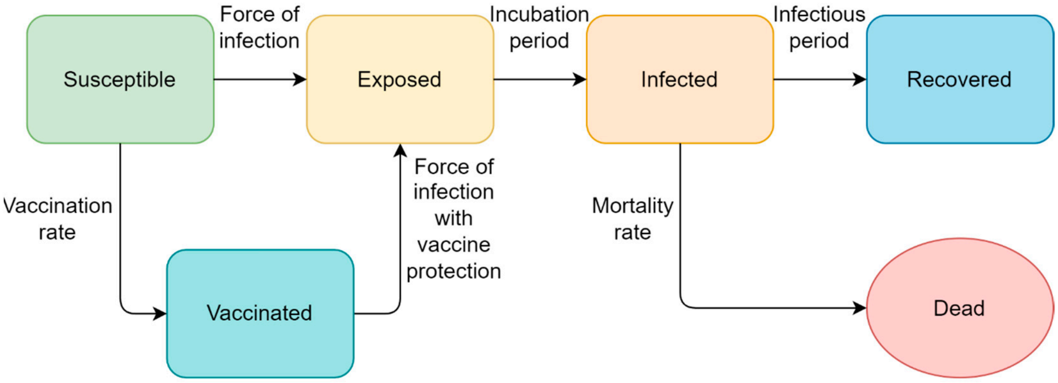

Figure S14. Diagrammatic representation of the SEIR model with vaccination used for analysis.

Calibration

The force of infection and case fatality rate in the model were calibrated so that the model fitted to the data on cumulative cases and deaths over the outbreak period. Figure S15 shows the model fit to the empirical data for the outbreak. The blue area represents the range of model simulations, and the blue line represents the median of all simulations, while the black dots represent the data. The range of model outcomes is driven by the stochastic sampling of compartment transitions at each time step, and uncertainty in model parameters. Although epidemiological data was not reported with regular frequency (only cumulative numbers at the end of the outbreak), the Pacific Public Health Surveillance Network issued daily updates of the outbreak status ('increasing' or 'decreasing or ongoing')[49]. This meant that in addition to fitting the model to cumulative cases and deaths at the end of the outbreak, the daily number of cases in the model could be calibrated to begin decreasing in October 2014 as was reported (represented by the vertical dashed line in panel three of Figure S15) . Finally, in order to match the reported vaccine doses administered by the ORI program, vaccine coverage in the model was linearly increased from an initial 38% to approximately 52% over the course of the outbreak.

Scenarios

The model scenarios implemented include:

- Baseline: The simulated outbreak, fit to empirical data;
- No ORI: A no ORI counterfactual, in which all vaccine delivery during the outbreak is set to 0;

- Increased vaccine delivery rate: The same total number of vaccine doses delivered, but the daily number delivered is 10% or 20% higher from the start of the ORI response (i.e., the final coverage is reached sooner). Referred to as '10% faster' and '20% faster' scenarios;

- Earlier response: ORI vaccine delivery begins a month earlier;

- Higher baseline vaccine coverage: A relative 10% and 20% increase of initial vaccine coverage before the outbreak begins. Referred to as '+10% coverage and '+20% coverage scenarios;

- Time varying ORI: Time-varying vaccine rollout, in which the response is either initially rapid and progressively slows over time, or initially slow and progressively increases over time (while still delivering the same number of total doses). Referred to as 'Faster start' and 'Slow starter' scenarios.

Model outputs
The model estimates cases and deaths over time, which were converted to disability-adjusted life years (DALYs) using the life expectancy, average age of death, and disability weight parameters defined in Table S5. Total economic costs were calculated from a societal perspective, including the direct healthcare costs as well as the cost of life years lost (the indirect costs). Direct healthcare costs are calculated by assuming that 25% of cases are severe and require hospitalisation, which incurs costs for in-patient care and treatment for approximately five days[47], the other 75% of cases are mild enough to not require hospital time and are managed with vitamin A supplementation and 20 minutes of healthcare provider time (assumed time for a visit with a physician or nurse at a clinic). The cost of life years lost were calculated from DALYs by multiplying by the statistical value of a life year. DALYs from future years of life lost are discounted at 3% per annum, and costs have been inflated and are presented in 2022 USD.

Multiple stochastic simulations were run for each scenario (n=100), and the reported values and uncertainty intervals represent the median and central 90-percentiles of simulated outcomes. Scenario impacts were calculated using a bootstrapping procedure to sample from the sets of results and estimate the median differences in cases, deaths, DALYs, and economic costs compared to the baseline model outcomes.

Results
Over April 2014-March 2015 period, the baseline model scenario resulted in 5,060 (4,952-5,110) diagnoses, and 29 (28-31) deaths (Figure S15), which translates to 841 (806-888) DALYs and a societal cost of $4,633,052 ($4,213,144-$5,155,208).

In the "No ORI" counter-factual scenario, there were 7,796 (7,342-8,398) diagnoses, and 43 (39-46) deaths, 1,240 (1,129-1,355) DALYs and $6,774,886 ($6,218,763-$7,341,299) in economic costs.

Compared to the baseline scenario, without the ORI program there were an additional 2,667 (2,432-2,900) cases, 14 (12-16) deaths, 402 (344-460) DALYs, and $2,238,008 ($1,931,824-$2,535,783) in health and economic costs. This accounts for an increase of 53% (48%-57%) in cases, 48% (40%-54%) in deaths, 48% (40%-56%) in DALYs, and 49% (42%-56%) in economic costs observed. These impacts (and the impacts observed in the other scenarios) are shown in Figure S16 and Figure S17, and Table S6 and Table S7.

The outcomes from the scenarios exploring the potential impacts of additional investment in the ORI program estimate that:

- Baseline vaccine coverage was the variable that had greatest influence, with a 10% or 20% relative increase in vaccine coverage before the outbreak reducing overall cases by 40% or 64%, and deaths by 42% or 66%, respectively;

- When the vaccine rollout was modelled as initially rapid and progressively slowing over time, there were 22% fewer cases and 19% fewer deaths. When the rollout was modelled as initially slow and progressively increasing over time, there were 17% more cases and 18% more deaths;

- When the ORI response was modelled as starting a month earlier, there were 12% fewer cases and 14% fewer deaths;

- A 10% increased vaccine delivery rate would have reduced cases by 7% and deaths by 5%, and a 20% increase to the vaccine delivery rate in the model reduced the outbreak size by 9% for cases and 8% for deaths, relative to the baseline outcomes.

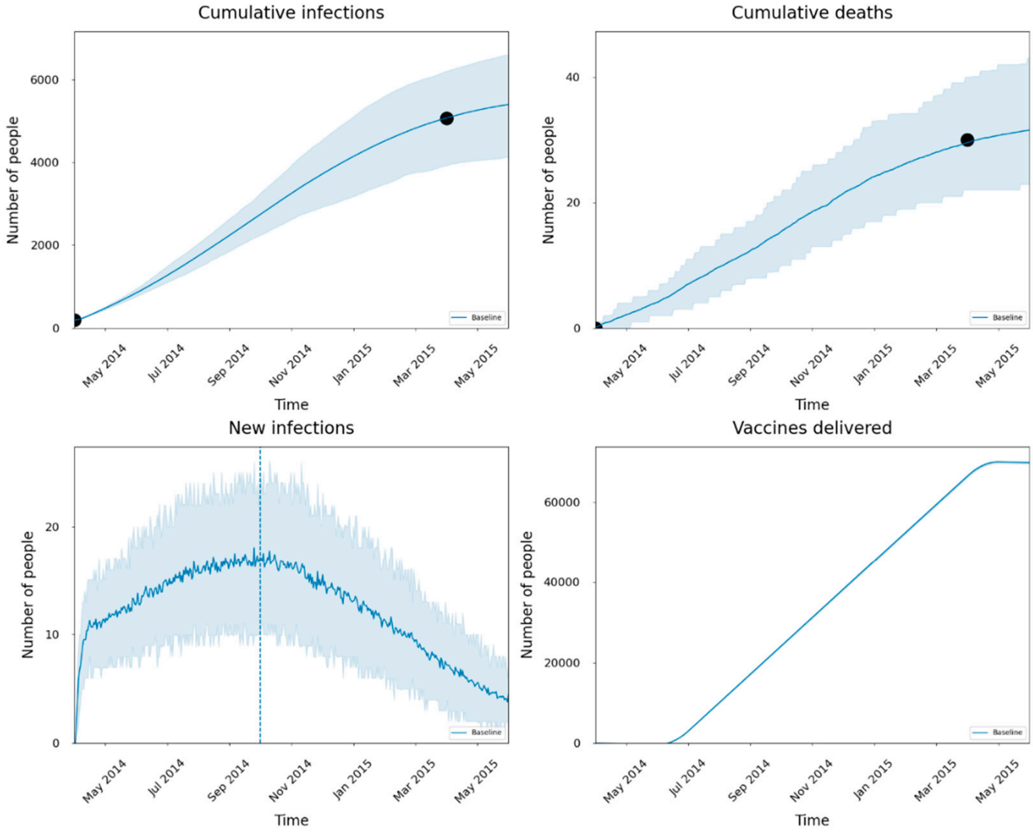

Figure S15. Calibration outputs for measles outbreak in Madang Province, PNG 2014-2015. Available epidemiological data were cumulative cases and deaths (black dots), however the Pacific Public Health Surveillance Network reported that the status of the outbreak transitioned from 'increasing' to 'decreasing or ongoing' in late October 2014 (denoted by the dashed line in the new infections plot) [49].

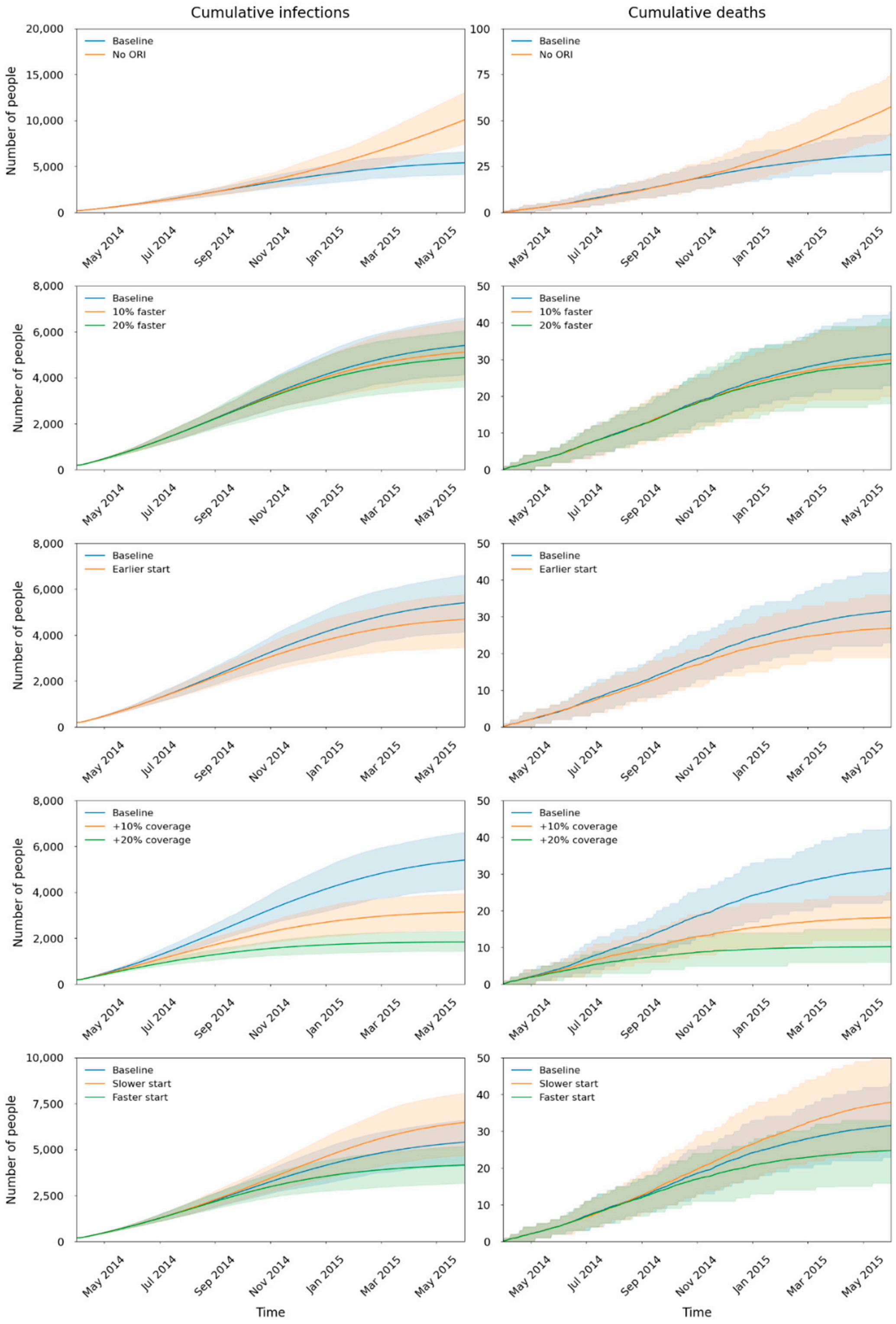

Figure S16. Timeseries outputs of infections and deaths for each scenario type compared to the baseline scenario.

Table S6. Relative impacts of each scenario on cumulative infections, deaths, DALYs, and costs over April 2014-March 2015 compared to the baseline scenario. Baseline results are presented as absolute numbers, for reference. Impacts are calculated using a bootstrap procedure to estimate the median values and 90% confidence intervals.

Scenario comparison	Difference in cases	Difference in deaths	Difference in DALYs	Difference in costs
Baseline cumulative outcomes	*5,060 (4,952, 5,110)*	*29 (28, 31)*	*841 (806, 888)*	*$4,598,176 ($4,438,799, $4,793,501)*
No ORI	53% (48%, 57%)	48% (40%, 54%)	48% (40%, 56%)	49% (42%, 56%)
10% faster	-7% (-9%, -5%)	-5% (-10%, -2%)	-6% (-9%, -2%)	-5% (-8%, -2%)
20% faster	-9% (-13%, -6%)	-8% (-13%, -2%)	-8% (-13%, -3%)	-8% (-12%, -3%)
Earlier response	-12% (-14%, -10%)	-14% (-20%, -10%)	-14% (-18%, -10%)	-13% (-17%, -10%)
+10% coverage	-40% (-41%, -39%)	-42% (-44%, -39%)	-42% (-45%, -39%)	-41% (-43%, -40%)
+20% coverage	-64% (-65%, -64%)	-66% (-68%, -64%)	-66% (-68%, -64%)	-66% (-68%, -65%)
Faster start	-22% (-24%, -20%)	-19% (-23%, -14%)	-18% (-22%, -15%)	-19% (-22%, -16%)
Slower start	17% (13%, 22%)	18% (12%, 25%)	18% (12%, 24%)	18% (13%, 22%)

Table S7. Absolute difference in outcomes for each scenario compared to baseline. Differences are calculated using a bootstrap procedure to estimate the median values and 90% confidence intervals.

Scenario comparison	Impact on cases	Impact on deaths	Impact on DALYs	Impact on costs (USD)
No ORI	2,667 (2,432, 2,900)	14 (12, 16)	402 (344, 460)	2,238,008 (1,931,824, 2,535,783)
10% faster	-352 (-439, -253)	-2 (-3, -1)	-47 (-80, -15)	-248,393 (-388,945, -111,189)
20% faster	-476 (-656, -319)	-2 (-4, -1)	-67 (-112, -26)	-358,587 (-562,962, -148,169)
Earlier response	-630 (-714, -512)	-4 (-6, -3)	-118 (-158, -78)	-615,645 (-794,452, -443,162)
+10% coverage	-2,028 (-2,077, -1,989)	-12 (-13, -11)	-352 (-386, -319)	-1,910,640 (-2,066,896, -1,776,505)
+20% coverage	-3,255 (-3,301, -3,199)	-19 (-21, -18)	-556 (-599, -520)	-3,030,085 (-3,171,384, -2914676)
Faster start	-1,127 (-1,224, -1,011)	-5 (-7, -4)	-155 (-199, -118)	-887,229 (-1,049,051, -730,042)
Slower start	882 (674, 1,118)	5 (4, 7)	149 (105, 198)	814,019 (613,425, 1,015,921)

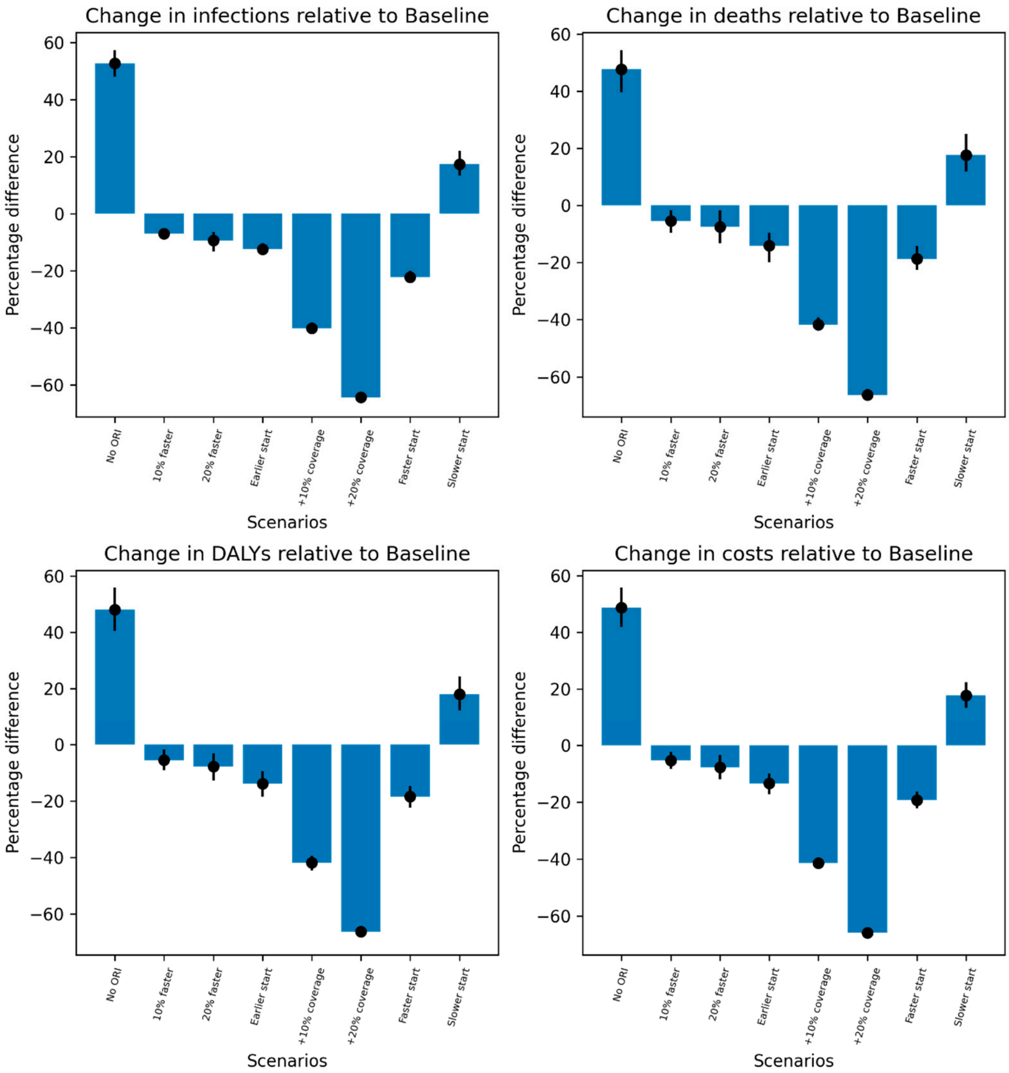

Figure S17. Relative difference in cumulative infections, deaths, DALYs, and costs in each scenario

Discussion

This modelling of the 2014 measles outbreak in Madang Province, PNG, estimates that the implemented immunization response had a significant impact on the size of the outbreak, compared to a counterfactual scenario with no ORI response. We estimate that the US$454,292 (calculated from Kamac et al[34].) spent on the vaccine response during the outbreak averted 2,667 cases and 14 deaths over the analysis period (01 April 2014 - 01 March 2015), however this is likely a considerable underestimate as with no immunization response the outbreak would likely have continued to grow beyond the modelled time period. These averted outcomes are estimated to translate into US$2·2M saved by the response, which is almost a five-fold return on investment.

From the examined results and sensitivity analyses, the parameter that influenced the model the most was the proportion of the population who were vaccinated in the first few months of the outbreak (impacted by either the underlying immunization coverage or the delivery rate at the start of the response). This strongly supports the case for increasing vaccine coverage in the absence of cases, and the model estimates that even a 10% relative increase in baseline vaccine coverage could save an additional US$1·9 million.

There were important but relatively smaller impacts observed for the scenarios with 10% or 20% accelerated vaccine rollout, a one-month earlier initiation of the vaccine response, or time-varying vaccine delivery schedules. It is unclear how much investment might be required to achieve the efficiency gains modelled in these scenarios, however the estimated impacts in Table S7 (particularly the economic impacts) could be used to inform willingness to spend thresholds for stakeholders. For example, the model estimates that a one-month earlier response could potentially save an additional US$615,000, and a 20% faster rollout save an additional US$360,000. These results could be used to motivate funding allocation to improve outbreak response plans and training, healthcare worker readiness, the cold chain, and logistical efficiencies.

There is a significant degree of uncertainty in the results of this analysis, which is illustrated in the wide uncertainty bands (Figure S16 and Figure S17, which represent the central 90-percentile results). This represents the range of outcomes that are possible when simulating a stochastic process with a low number of cases.

Conclusion
The presence of the ORI program in the 2014-15 Madang Province measles outbreak had a significant dampening effect on the outbreak, and without the program there were estimated to have been an additional 2,667 cases (+53%), 14 deaths (+48%) and US$2·2 million in societal economic costs (+48%), with a 4·8-fold return on investment. Increasing baseline vaccine coverage should remain the highest priority, and the results suggest that early and rapid responses can have the greatest impacts.

Supplement G: Detailed 2020 Equateur Province, Democratic Republic of the Congo Ebola analysis

This section details the analysis performed for the Ebola outbreak in Equateur Province, Democratic Republic of the Congo (DRC). The objectives were to estimate the return-on-investment of the ORI program, in a way that is useful for government and international stakeholders to support continued financing of these programs in DRC.

Background
In June 2020 the eleventh Ebola outbreak was declared in DRC and remained active until November. The outbreak caused a total of 130 reported infections, all of which occurred in Equateur Province, which will be the focus of this study[35]. 55 deaths were recorded among the cases, and during the outbreak there were approximately 43,000 vaccine doses administered[50].

The objectives of this analysis were to estimate the return-on-investment of the ORI program, in a way that is useful for government and international stakeholders to support continued financing of these programs in DRC. The analysis also considered the impacts of targeted improvements to key aspects of the response, to identify the highest priority elements of the response (e.g., speed versus targeted coverage) and where the greatest gains could be during a future Ebola outbreak.

Aims
Aim 1: estimate the cumulative infections, deaths, disability adjusted life years (DALYs), and economic costs averted by the ORI program.

Aim 2: estimate the impact of the ORI on the risk of the outbreak exceeding 200 cases

Aim 3: identify elements of the vaccine response (speed of rollout, targeting, time to commencement) that have the greatest influence on ORI program impact.

Methods

Data

A range of relevant data were available for this setting, including: the start and end dates of the immunization response, the time taken between diagnosis of the first case and the response beginning, the total number of cases, deaths, and vaccine doses delivered over the outbreak period, the number of contacts traced as a part of the response, and the number of contacts vaccinated as a part of the response[35,50].

Model

To evaluate outbreak risk and contact tracing an agent-based model was required. We used the Covasim model[51], but adapted for Ebola transmission and prognoses (represented schematically in Figure S18 and Figure S19). Agents in the model are assigned an age (which affects infection outcomes) and are assigned household contacts and community contacts. Additionally, agents can receive vaccines with characteristics matching the Ervebo vaccine which was used during the outbreak[52]. Both susceptible and vaccinated people can become infected at a rate that is proportional to dynamic prevalence, however vaccinated people have reduced risk due to vaccine protection. Following infection, people have an incubation period before becoming infectious, and after a period some infected agents will progress to more severe disease. Those who do not advance to a more severe disease state will recover, whereas those with severe disease can either die or recover. As Ebola can still be transmitted via contact with a corpse, the model includes a transmission pathway until burial occurs, however for people identified as Ebola cases a proportion of burials are assumed to be handled safely, which removes this risk of transmission. The model's progression and transmission pathways are based on structures used in other modelling studies[53].

The duration of vaccine immunity was set to well beyond the scope of the model period as it is assumed that no waning of immunity effects would be relevant over the outbreak period. Table S8 details the parameters used within the model.

Agents within the model are assumed to seek a test when symptomatic, with a probability of 2·5% per day that they are symptomatic; due to increased awareness as the outbreak progresses, this probability increases to 5% per day after one month, 10% per day after two months, and 20% per day after 3 months.

Contact tracing captures 100% of household contacts within 1 day, and 90% of community contacts in 1-2 days. These values are assumptions, calibrated to capture the high numbers of contacts which are reported to have been successfully traced over the outbreak[35]. Traced contacts are required to quarantine for 21 days, with PCR tests on day 1 and day 21. It is unclear how effective the notification of contacts was, as a WHO report indicates that in August-September 2020 only 81% of contacts were successfully notified and over the duration of the outbreak 67% of detected cases had not previously been identified as contacts[35].

Table S8. Model parameter values and sources.

Parameter	Value	Source
Susceptibility to infection	Varies with age	Based on values from Table 4 in: Bower et al[54].
Probability of developing severe disease	70%	Proportion of cases which progressed to Stage 2 or 3 disease from Kangbai et al[55].
Probability of death, given severe disease	Varies with age	Based on values from Supplementary Appendix 1 of WHO Ebola Response Team[56].
Average duration of exposed period	12·7 days	Haas CN[57].
Average duration of mild infection	10·0 days	Assumption based on ranges and reported means in Kadanali and Karagoz[58], Singh and Ruzek[59], Legrand et al.[60], and Simpson DI[61].
Average time to develop severe disease	6·0 days	Assumption based on reported 5–7-day ranges for time to develop fever and rash in Kadanli and Karagoz[58], and Goeijenbier et al[62].
Average duration of severe infection (survived)	10·4 days	Mean time in hospital for survivors from Hartley et al[63]. Aligns with convalescence time for survivors from Simpson DI[61].
Average duration of severe infection (died)	1·5 days	Mean time to death after symptom onset is 7·5 days from Centers for Disease Control and Prevention[64]. Subtract 6 days for average time to develop severe disease.
Average time to burial (unsafe funeral)	2·0 days	Assumption based on parameter used in Legrand et al[60].
Vaccine protection	97·5%	Based on reported value in World Health Organization[52].

against infection		
Equateur Province population by age		United Nations, Department of Economic and Social Affairs, Population Division[65]. DRC population by age scaled to the Equateur population size of 1,712,000.
Equateur Province household size distribution		United Nations, Department of Economic and Social Affairs, Population Division[66]. DRC household size distribution scaled to the Equateur population size of 1,712,000.
Number of doses delivered during response	43,000	World Health Organization[35]. The rate of delivery is assumed increase each month for the first two months, and decrease over the latter three months.
Cost of response	US$ 2,067, 427	Gavi, the Vaccine Alliance[67]
Disability weights for Ebola infection	Acute: 0·133 Chronic: 0·219	Global Burden of Disease Collaborative Network[68]
Average life expectancy in DRC	60 years	The World Bank[69]
Ebola PCR test sensitivity	99%	Assumption, as the GeneXpert PCR tests seem to be considered the gold standard test.
Ebola PCR test delay	1 day	Assumption.
Statistical value of a life year	US$918·23	Calculated as 1·75 times the Gross Domestic Product (GDP) per capita for DRC, based on Stenberg et al[48].

*Inflated to 2022 US$. Source does not contain costing breakdown.

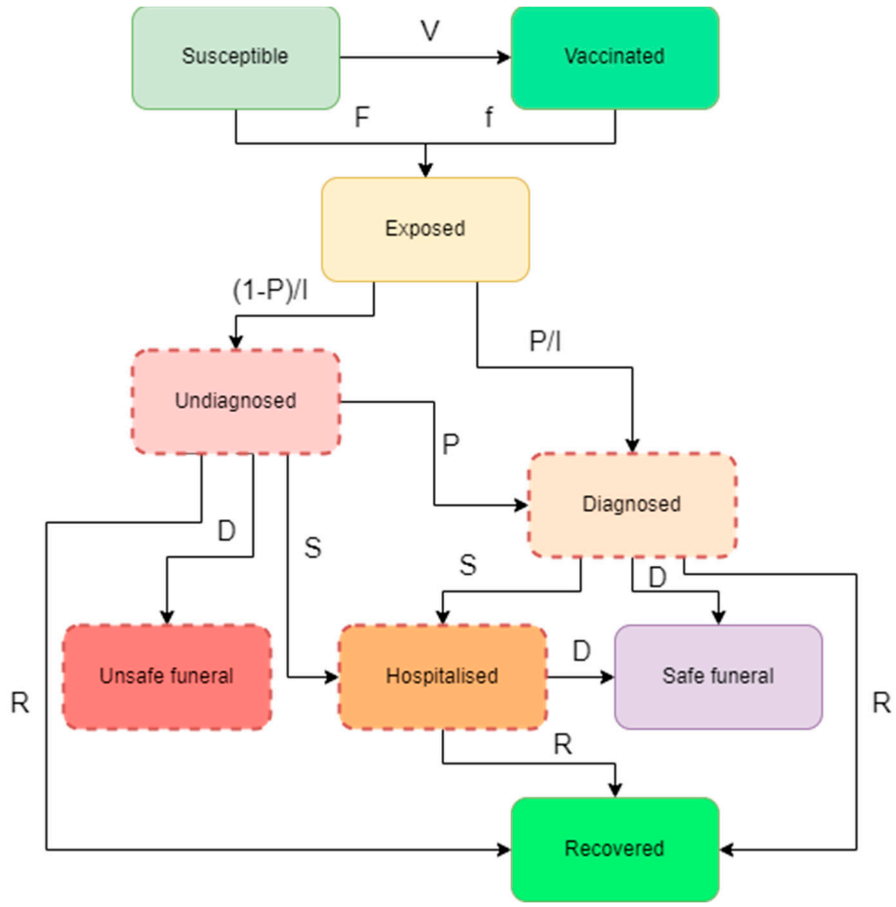

Figure S18. Diagrammatic representation of the Ebola disease states and progression used for analysis. V is the vaccination rate, F and f are the force of infection for vaccinated and unvaccinated agents, respectively, P is the probability of getting a test when symptomatic, I is the incubation period, S is the rate of developing severe disease, D is the mortality rate, and R is the recovery rate. States which have a dashed outline are infectious in the model, while states with a solid outline are not infectious.

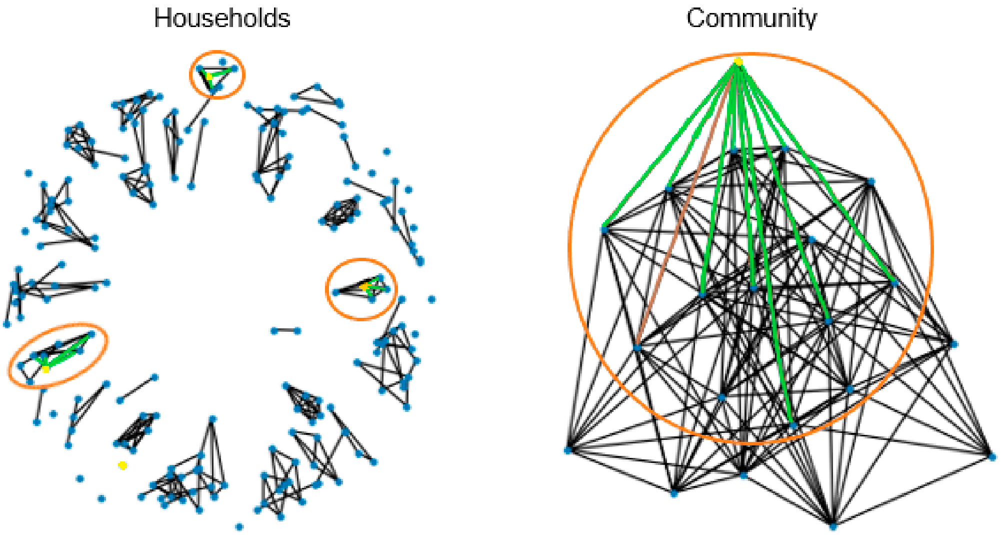

Figure S19. Example contact network structures between in the model. Each dot represents an agent, and each line a contact interaction. Yellow agents are infectious and

identified by symptomatic testing, with green and brown lines representing successfully and unsuccessfully traced interactions, respectively. Successfully traced contacts are prioritised for vaccination when a ring-based vaccination strategy is used.

Calibration

For the calibration the model was initialised with a population of 100,000 agents. The overall transmission risk per contact (in household and community settings), the per-day probability of a symptomatic individual seeking testing, the probability of contacts being successfully traced, the number of seed cases, and the infection seeding date were varied such that the distribution of model outcomes for diagnoses, deaths, total contacts traced, and the number of contacts vaccinated was centred on the reported empirical data. Figure S21 shows the model fit to the empirical data for the outbreak. The blue area represents the range of model simulations, and the blue line represents the median of all simulations, while the black dots represent the data. The range of simulation outcomes is driven by the stochastic nature of the agent-based model, with the initialisation conditions randomising where infections are seeded and how contact networks are generated. The epidemiological data that the model was aligned to was reported with irregularity in USAID situation reports and WHO disease outbreak reports[35,50]. Finally, the reported vaccine doses administered by the ORI program in the model were matched to the total doses reported by the WHO[35].

Figure S20 shows how the accepted simulation seeds compare to the diagnoses and deaths data. Simulations that diverged from the actual case or death data by more than 15% of the cumulative values (beyond an initial growth period from June-September) were rejected from further analysis. Based on these filtered simulations we extracted a distribution of the overall transmission risk per contact parameter value within the model, which is a key driver of model outcomes. This distribution was used to sample the transmission risk used in each of the n=2300 simulations run for each scenario. Figure S21 shows the spread of the calibrated simulation runs used to estimate parameters for this study. The variation between simulations is due to stochasticity within the model, particularly when case numbers are relatively low and the outcomes of each individual case therefore have a significant impact on the trajectory of the outbreak.

Scenarios

The model scenarios implemented include:

- Baseline: The simulated outbreak, fit to empirical data;

- No ORI: A no ORI counterfactual, in which all vaccine delivery during the outbreak is set to 0;

- Increased vaccine delivery rate: The number of vaccines delivered is 10% or 20% higher from the start of the ORI response until October 2020, when the final case was detected. Referred to as '10% faster' and '20% faster' scenarios;

- Earlier response: ORI vaccine delivery begins seven days earlier;

- Non-targeted vaccines: Vaccines are delivered randomly to the population, rather than prioritising known contacts of cases, which is assumed to occur in all other scenarios;

- Vaccine efficacy sensitivity: The Ervebo vaccine's protection against infection (as detailed in Table A8) is based on limited data, and as such sensitivity analyses were undertaken to assess the impact of the vaccine's efficacy on model outcomes;

- Contact tracing sensitivity: The effectiveness of the contact tracing and follow-up on household and community contacts is based on unvalidated assumptions, so we undertook sensitivity analyses to understand how these assumptions impacted the model outcomes.

Model outputs

The model estimates cases and deaths over time, which were converted to disability-adjusted life years (DALYs) using the life expectancy and disability weight parameters defined in Table S8. Total years of life lost are calculated by tracking the age of death of agents during model simulations. Total economic costs were calculated from a societal perspective, including the direct healthcare costs as well as the cost of life years lost (the indirect costs). Direct healthcare costs are calculated using the same methodology as Bartsch et al.[13], which uses an ingredients-based approach and divides the levels of care provided into either supportive or extensive depending on the patient's symptoms. We assume that 20% of patients require extensive care based on how the symptom definitions used by Bartsch et al. align with symptom frequencies reported in the literature[29,57,70]. The cost of life years lost were calculated from DALYs by multiplying by the statistical value of a life year. As Ebola can cause ongoing complications in some survivors, we considered the impact of disability from acute infection and assumed chronic infection occurred in 70% of cases and lasted for one year[71-73]. DALYs from future years of life lost are discounted at 3% per annum, and costs have been inflated and are presented in 2022 USD.

Multiple stochastic simulations were run for each scenario (n=2300), and the reported values and uncertainty intervals represent the median and central 90-percentiles of simulated outcomes. Scenario impacts were calculated using a bootstrapping procedure to sample from the sets of results and estimate the median differences in cases, deaths, DALYs, and economic costs compared to the baseline model outcomes.

Results

Over June-November 2020 period, the baseline model scenario resulted in a median 175 (98-323) infections, and 56 (30-102) deaths, which translates to 1578 (863-2793) DALYs and a societal cost (including the direct cost to the healthcare system from disease burden and the broader impacts to the economy due to accrued mortality and morbidity in the population) of US$1,448,144 (US$773,832-US$2,521,231).

In the "No ORI" counter-factual scenario, there were a median of 216 (104-412) infections, and 69 (34-127) deaths, 1915 (953-3526) DALYs and US$1,758,546 (US$890,390-US$3,312,873) in economic costs.

Compared to the baseline scenario, without the ORI program there were an additional 41 (20-87) infections, 13 (6-27) deaths, 338 (150-711) DALYs, and US$309,636 (US$134,534-US$637,127) in health and economic costs. This is an increase of 23% (20%-27%) in cases, 23% (19%-27%) in deaths, 21% (17%-25%) in DALYs, and 21% (17%-25%) in costs observed. These impacts (and the impacts observed in the other scenarios) are shown in Figure S22, Figure S25, Figure S26, Figure S27, and Table S9 and Table S10. Additionally, as is represented in Figure S23 and Figure S24, the implementation of the ORI is estimated to have reduced the risk of the outbreak exceeding a threshold of 200 infections by 20 percentage points, from 56% to 36% of simulations.

The outcomes from the scenarios exploring the potential impacts of additional investment in the ORI program estimate that:

- The targeted delivery of vaccines to known contacts of cases was the factor that had greatest influence, with the ring-based vaccination captured in the baseline scenario reducing overall cases by 17% and deaths by 16% compared to the non-targeted vaccines scenario. The implementation of the targeted vaccination is estimated to have reduced the risk of the outbreak exceeding a threshold of 200 infections by more than 15 percentage points.

- When the vaccine rollout was modelled as starting a week earlier, or with a 10% or 20% faster rollout rate there was little impact on the median outcomes or proportion of simulations exceeding the 100, 150, or 200 infection thresholds compared to the baseline scenario. The stochastic effects lead to wide and overlapping confidence intervals with no significant difference across these scenarios.

- Exploring the results of the sensitivity analyses in Figure S28 indicates that the model is relatively sensitive to the assumption of the vaccine impact on onward transmission for breakthrough infections. Compared to the point estimate assumption of a 50% reduction in transmission, when no reduction in transmission was assumed, there were 12% more DALYs, and when a 100% reduction on transmission was assumed, there were 10% fewer DALYs.

- When examining the vaccine's efficacy against infection acquisition, we tested reductions to 95% and 90% protection from the 97·5% used in the model, which is the reported efficacy by the WHO.[46] These reductions had minimal impact on outcomes, resulting in 2-3% more DALYs.

- The model is also sensitive to assumptions around the contact tracing capabilities during the outbreak, however it requires a 50% relative reduction of the baseline tracing probabilities for household and community contacts before meaningful impacts on DALYs are observed. For example, reducing the probability that contacts are successfully traced by 50% relative to the baseline assumptions increases DALYs by 8%.

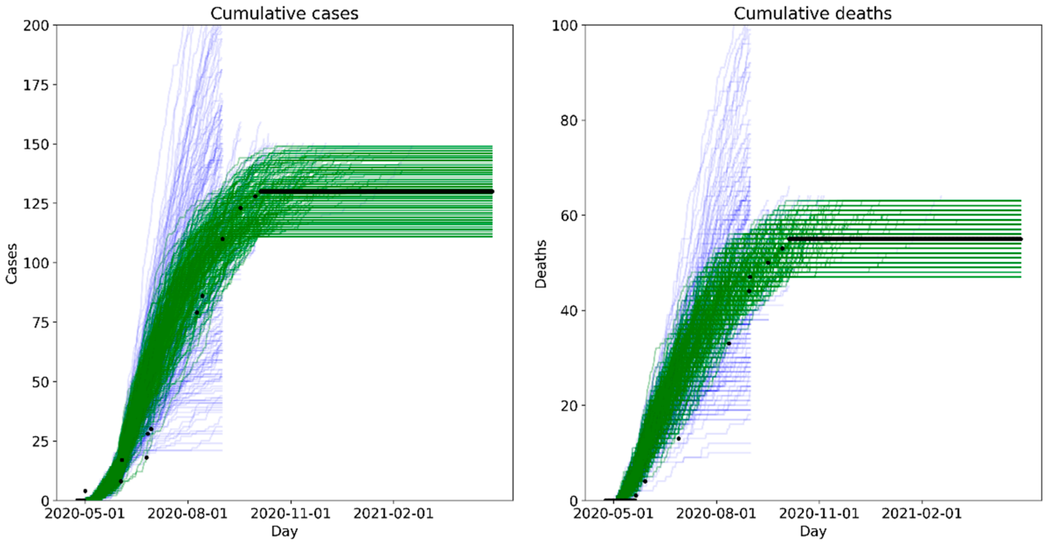

Figure S20. Individual model simulations of the Ebola outbreak. Differences between simulations are due to stochastic model effects, and some simulations (blue) are rejected from further analysis because they diverged from the actual data early on. The distribution of transmission probabilities from retained simulations (green) were used for subsequent scenarios.

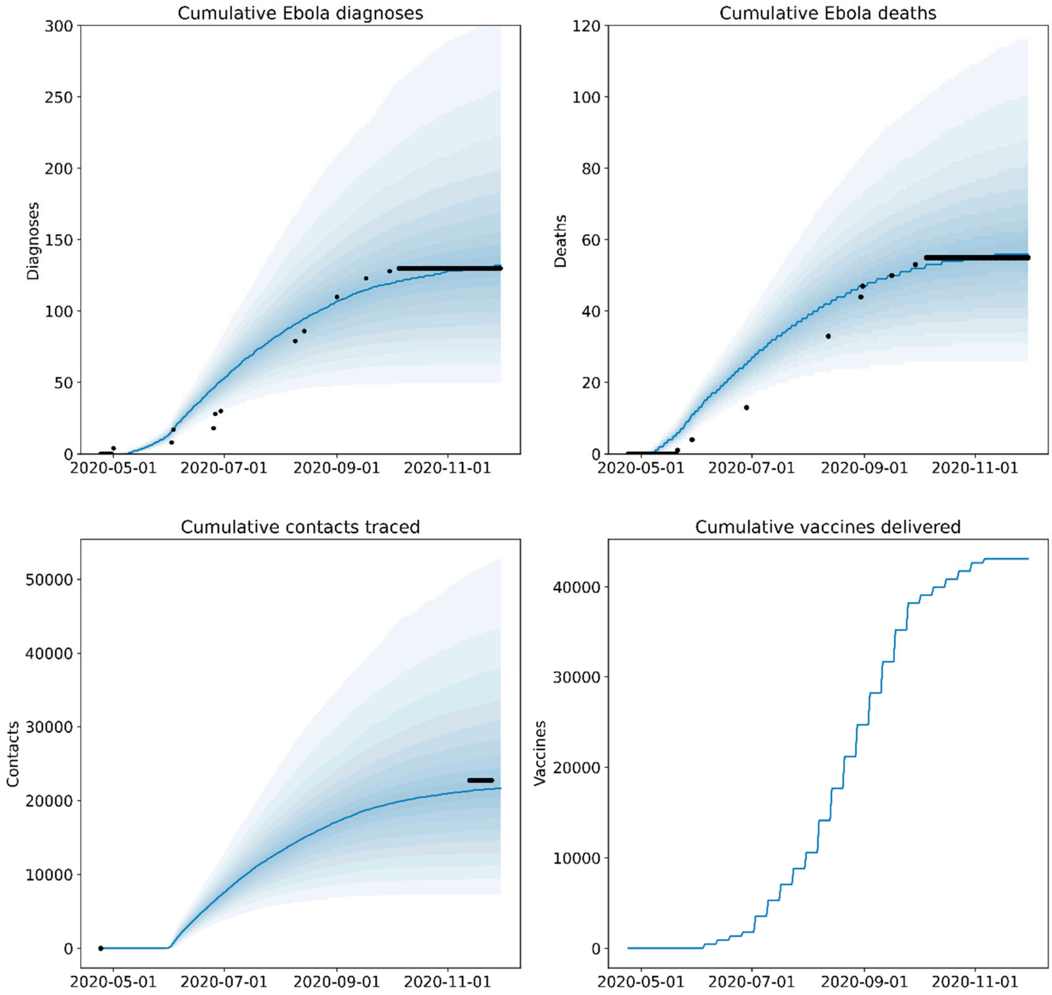

Figure S21. Calibration outputs for Ebola outbreak in Equateur Province, DRC 2020. Available epidemiological data were cumulative cases, deaths, and cumulative contacts traced (black dots). The transmission probability for each simulation was sampled from a distribution determined by the filtered simulations represented in indicates that the model is relatively sensitive to the assumption of the vaccine impact on onward transmission for breakthrough infections. Compared to the point estimate assumption of a 50% reduction in transmission, when no reduction in transmission was assumed, there were 12% more DALYs, and when a 100% reduction on transmission was assumed, there were 10% fewer DALYs.

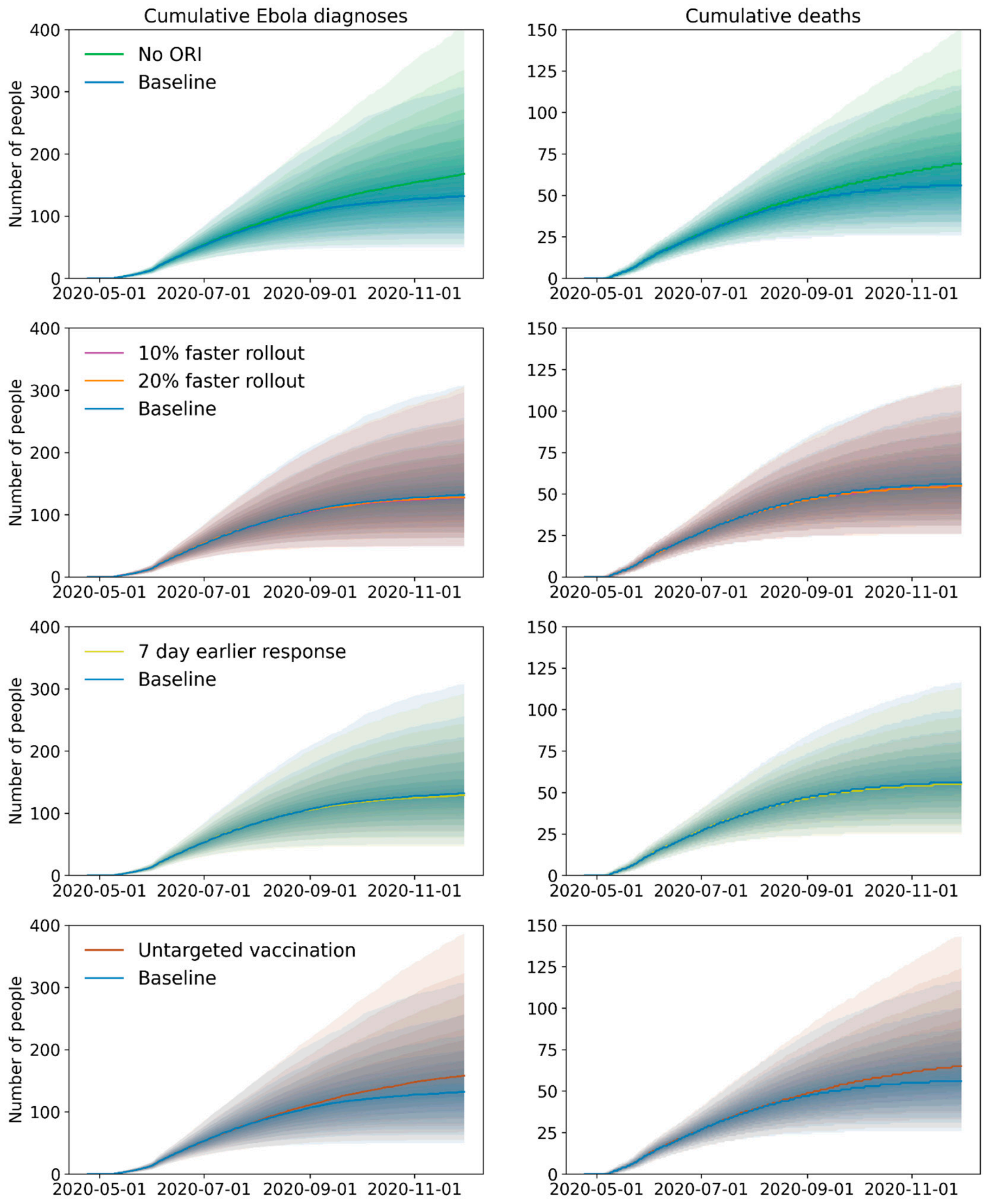

Figure S22. Timeseries outputs of infections and deaths for each scenario type compared to the baseline scenario.

Table S9. Relative impacts of each scenario on cumulative infections, deaths, DALYs, and costs over June-November 2020 compared to the baseline scenario. Baseline results are presented as absolute numbers, for reference. Impacts are calculated using a bootstrap procedure to estimate the median values and 90% confidence intervals.

**Scenario comparison**	**Difference in infections**	**Difference in deaths**	**Difference in DALYs**	**Difference in costs**
Baseline cumulative outcomes	*175 (98-323)*	*56 (30-102)*	*1578 (863-2,793)*	*US$1,448,144 (US$773,832-US$2,521,231)*
No ORI	23% (20%, 27%)	23% (19%, 27%)	21% (17%, 25%)	21% (17%, 25%)

10% faster	-3% (-5%, -1%)	-2% (-5%, 0%)	-2% (-5%, 0%)	-2% (-5%, 0%)
20% faster	-2% (-4%, 1%)	-2% (-5%, 0%)	-2% (-5%, 1%)	-2% (-5%, 0%)
Earlier response	-2% (-4%, 1%)	-2% (-5%, 2%)	-2% (-5%, 1%)	-2% (-5%, 0%)
Non-targeted vaccines	17% (14%, 21%)	16% (12%, 20%)	15% (11%, 19%)	15% (11%, 18%)

Table S10. Absolute difference in outcomes for each scenario compared to baseline. Differences are calculated using a bootstrap procedure to estimate the median values and 90% confidence intervals.

Scenario comparison	Impact on infections	Impact on deaths	Impact on DALYs	Impact on costs (USD)
No ORI	41 (20, 87)	13 (6, 27)	338 (150, 711)	309,636 (134,534, 637,127)
10% faster	-5 (-5, -2)	-1 (-2, 0)	-39 (-45, 2)	-34,409 (-39,781, 4,919)
20% faster	-3 (-4, 2)	-1 (-2, 0)	-34 (-42, 17)	-30,458 (-37,744, 12,153)
Earlier response	-3 (-4, 2)	-1 (-2, 2)	-31 (-41, 18)	-28,040 (-35,182, 12,133)
Non-targeted vaccines	30 (13, 67)	9 (4, 20)	231 (94, 522)	213,203 (84,881, 462,508)

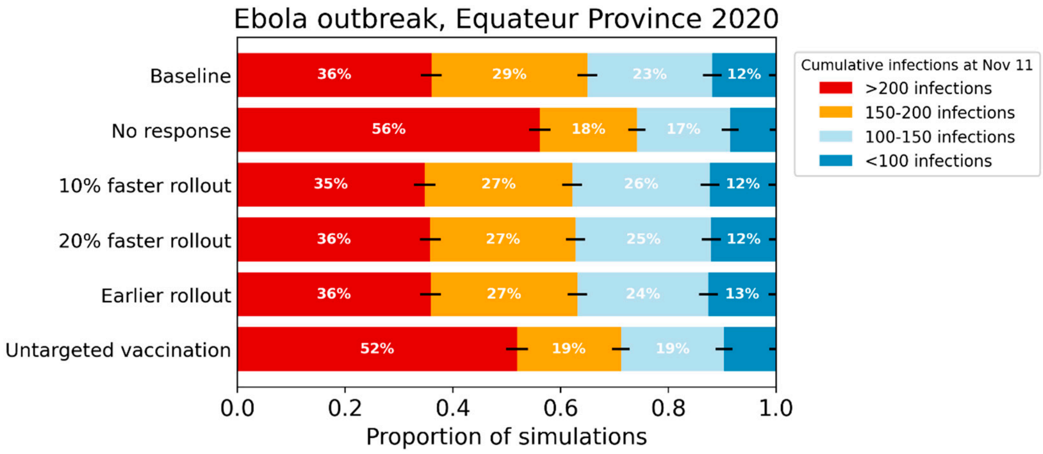

Figure S23. Proportion of simulations which exceeded thresholds of 100, 150, and 200 infections by November 11 2020, for each modelled scenario.

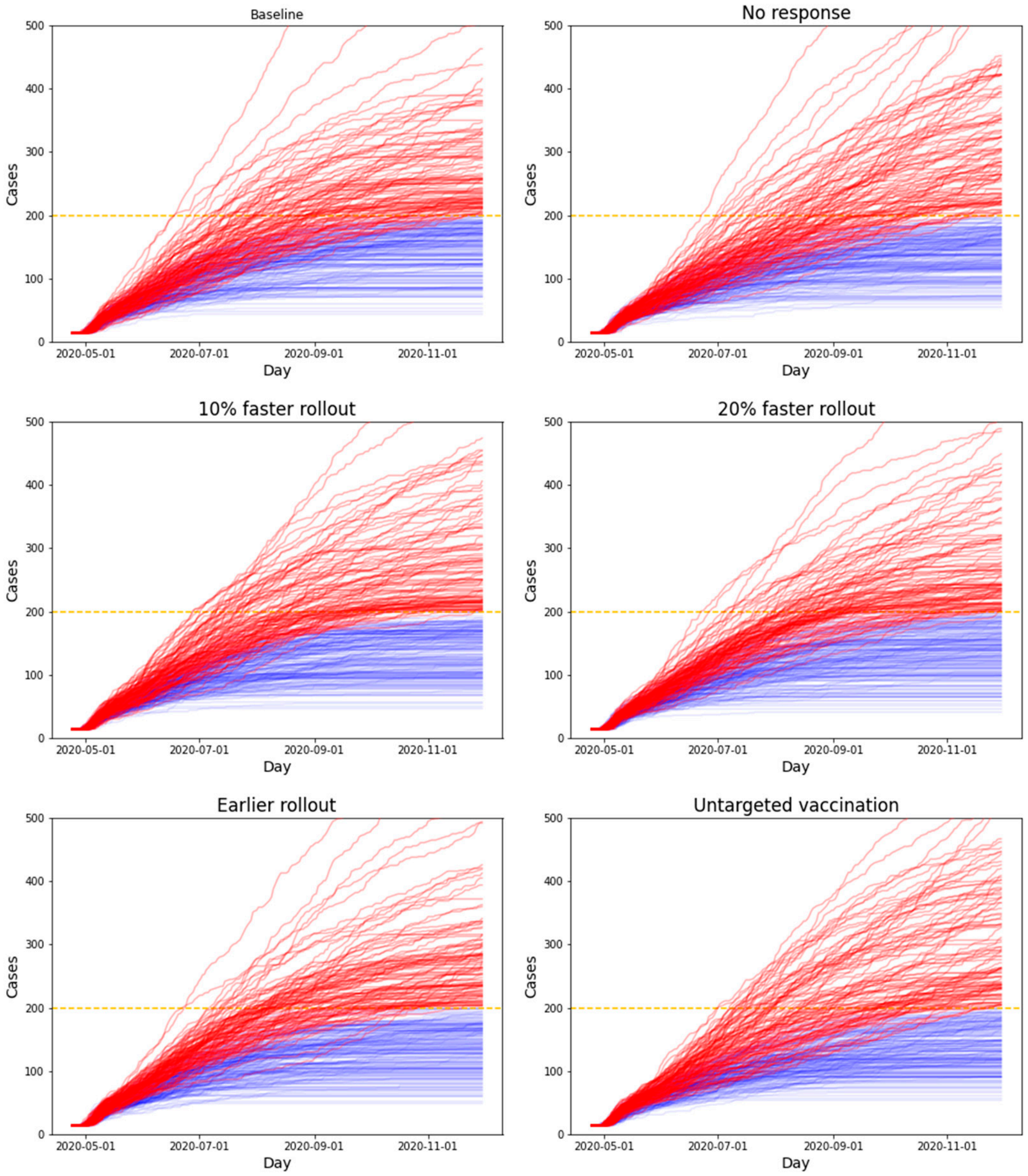

Figure S24. Time series plots of simulations for each modelled scenario, differentiated by whether cumulative infections exceeded (red) or did not exceed (blue) 200 infections.

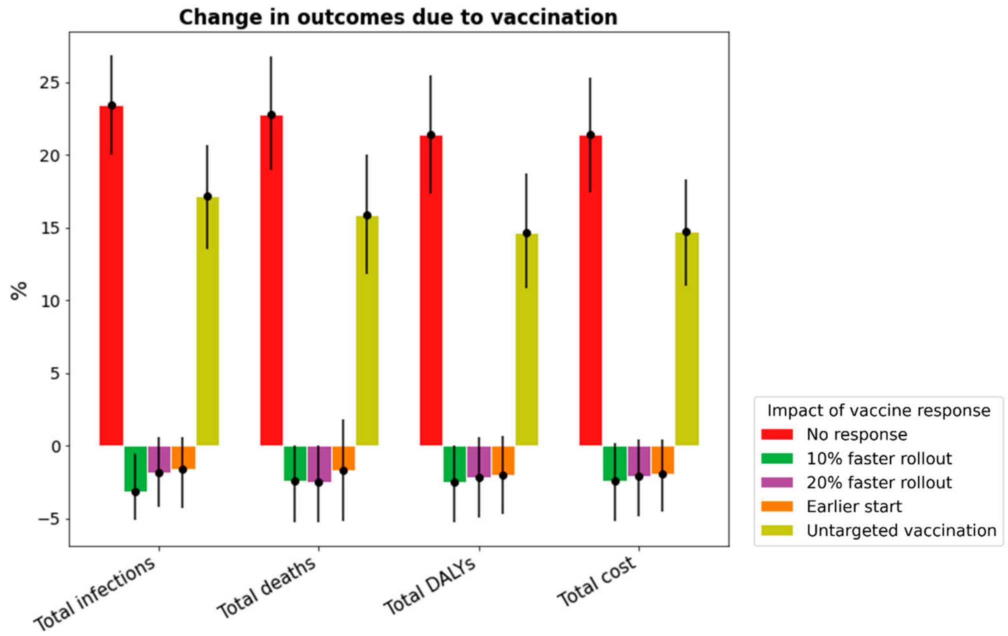

Figure S25. Relative difference in cumulative infections, deaths, DALYs, and costs in each scenario, compared to the baseline. The 10% faster rollout scenario produced slightly better infection outcomes than the 20% faster scenario, but this is a difference of two infections between their median observed impacts.

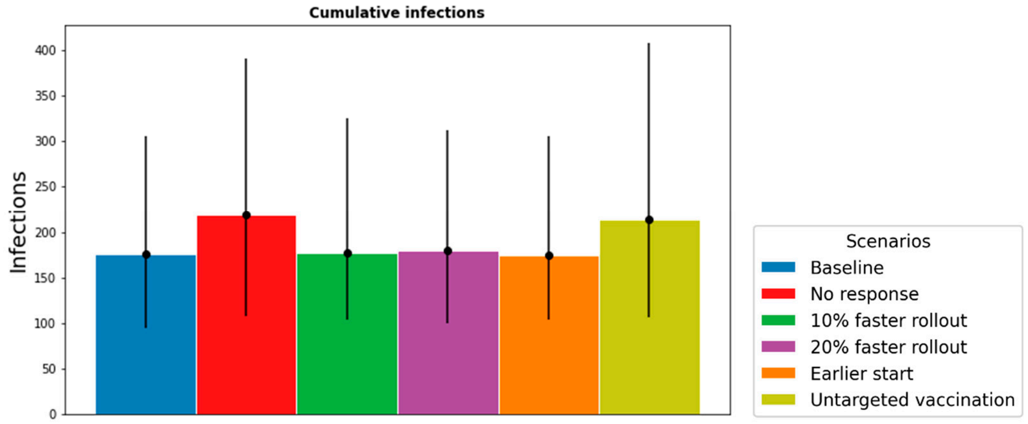

Figure S26. Cumulative infections by November 11 2020 for each scenario

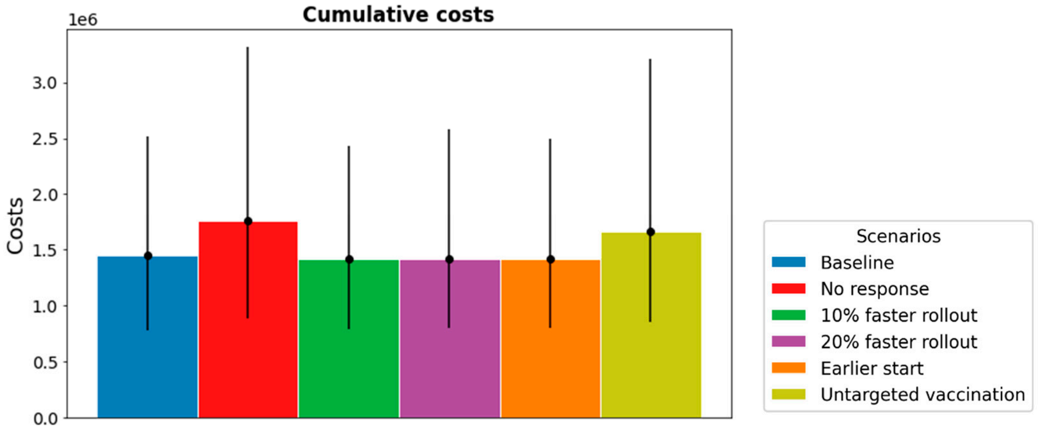

Figure S27. Cumulative cost of hospitalisations and monetized DALYs (in 2022 millions of US$) by November 11 2020 for each scenario

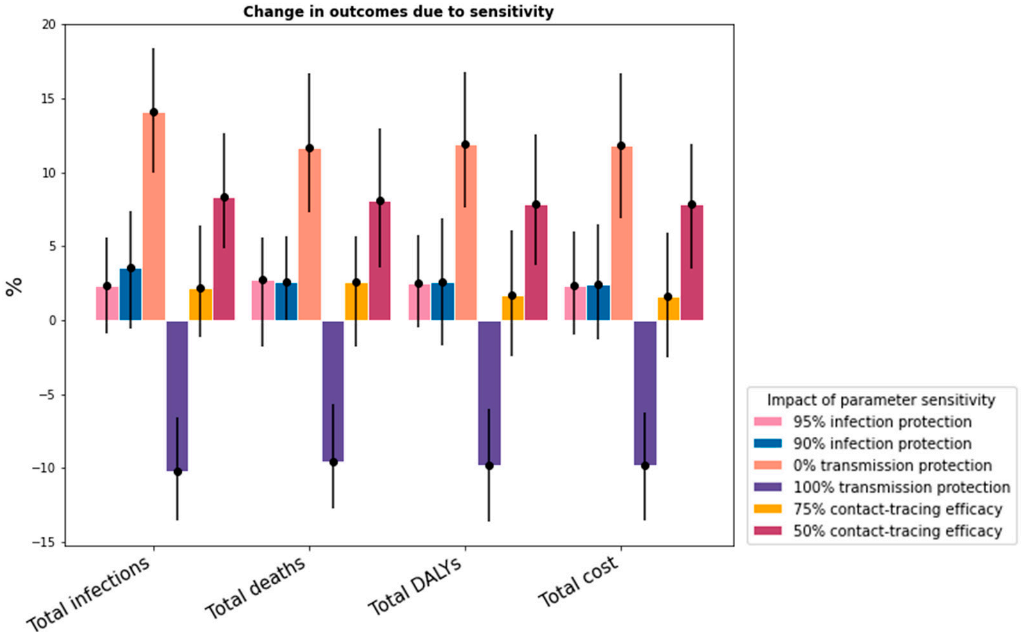

Figure S28. Relative difference in cumulative infections, deaths, DALYs, and costs in each sensitivity scenario, compared to the baseline

Discussion

This modelling of the 2020 Ebola outbreak in Equateur Province, DRC, estimates that the implemented immunization response had a significant impact on the size of the outbreak, compared to a counterfactual scenario with no ORI response. We estimate that the US$2.1M spent on the vaccine response during the outbreak[67], averted 41 cases and 13 deaths over the analysis period, however this is likely a considerable underestimate as with no immunization response the outbreak would likely have continued to grow beyond the modelled time period. These averted outcomes are estimated to translate into US$300K in direct health system and indirect societal costs saved by the response. In this setting, the economic benefits from the response are estimated to be significantly less than the cost of the response itself. A significant driver of this difference is the high cost per dose of the Ebola vaccine.[74]

The presence of the ORI program is estimated to have reduced the risk of the outbreak exceeding 200 infections by 20 percentage points. Based on the results of the modelled baseline scenario, an outbreak which exceeded 200 infections could have caused upwards of 64 deaths and US$1.6M in direct health system and indirect societal costs. This sits on the upper bound of costs estimated by Bartsch et al[29]. ($1,127 per case that recovers, and $23,474 per case that does not, inflated to 2022 USD), based on the large Ebola outbreaks in Guinea, Liberia, and Sierra Leone in 2014, which is estimated to have caused US$2·8B in economic damages[75]. The 2020 Equateur outbreak was the 11th Ebola outbreak in DRC, having been immediately preceded by the North Kivu outbreak (the second largest in recorded history)[76]. The North Kivu outbreak resulted in 3,481 cases and 2,299 deaths across 2018-2020[77], which translates into approximately US$55·4M using the estimates from Bartsch et al[29].

Gavi plans to spend US$150M on Ebola vaccines between 2021-2025[78], to protect vulnerable populations against outbreaks of Ebola, which can be expected to reduce

morbidity, mortality, and the risk of large, disruptive outbreaks. It is unclear what scale of outbreak could have occurred in Equateur, but clearly larger outbreaks result in significantly worse health and economic outcomes. During a large outbreak of Ebola, communities are heavily impacted by mortality, morbidity, and the restrictions implemented to reduce the spread of the disease. These impacts go beyond the direct and indirect health costs captured here, into broad and long-lasting decreases in employment, economic growth, and government spending[75,79].

From the examined scenarios, the targeting of vaccinations to contacts compared to vaccination at random had the greatest impact on outcomes, suggesting the ring-based vaccination strategy is one of the most critical elements of Ebola ORI programs. We found that on average, random allocation of vaccines led to 17% more infections, 16% more deaths, and produced a higher risk of the outbreak exceeding 200 infections compared to a ring-based vaccination strategy. An effective contact tracing program can find and quarantine known contacts of a case before they become infectious, and coupling with this with targeted vaccination can effectively disrupt chains of transmission. The key factors of this disruption are how quickly and effectively the contact tracing program can find and quarantine contacts and the degree of protection that vaccination imparts against both infection and onward transmission for breakthrough infections. For the 2020 Ebola outbreak in DRC there was limited data to inform inputs such as the estimated proportion of contacts which were successfully traced, the time taken to trace contacts, and the proportion of traced contacts who were successfully followed-up, and the model sensitivity to these assumptions suggests that collection of this information in future outbreaks would be beneficial.

Vaccination is only one component of the response to Ebola outbreaks. For example, public health responses, including testing, contact tracing, and quarantine can reduce transmission and have been able to end outbreaks before the vaccine was available. This is likely the driving factor behind the lack of impact observed in the 10% and 20% faster vaccine rollout and earlier vaccination initiation scenarios, and we see that the vaccines are most impactful when working synergistically with the contact tracing program rather that purely the speed of the vaccine delivery. This also explains the inconsistency observed in Figure S25, which shows that the 10% faster rollout scenario produced slightly better infection outcomes than the 20% faster scenario. In the early stages of the outbreak there are relatively fewer infections, and therefore relatively fewer contacts to trace and vaccinate. As such, additional vaccines delivered early are not necessarily delivered to traced contacts, which appears to be a significant driver of the ORI impact in this analysis. The outcomes from these scenarios are expected to be different if applied to a larger outbreak, or one with fewer vaccines available, but in the context of the 2020 Equateur outbreak there were approximately 43 thousand vaccines delivered and 135 cases detected, so there were likely ample doses available to target the traced contacts of each case.

There is a significant degree of uncertainty in the results of this analysis, which is illustrated in the wide uncertainty bands seen in Figure S22 and Figure S24, which represent the central 90-percentile results. This range of outcomes is expected, and is indicative of how much of a driver stochastic variation is within the model, especially for an outbreak like this with relatively few cases. There is additional uncertainty inherent in the assumptions which inform key drivers of the model, such as the contact tracing efficiency and vaccine parameters, and more data to inform these aspects would help to reduce uncertainty in the model.

Conclusion

The presence of the ORI program in the 2020 Equateur Province Ebola outbreak had a

dampening effect on the outbreak, and without the program in place there were estimated to have been an additional 41 cases (+23%), 13 deaths (+23%), US$300 thousand in societal economic costs (+21%), and to have reduced the risk of the outbreak exceeding 200 cases from 56% to 36%. Effective contact tracing and implementation of ring-based vaccination strategies should remain a priority, as our results indicate they were impactful in this setting.
